# Enzyme Immobilization by Hydrogen‐Bonded Organic Framework for the Construction of Plant‐Wearable Sensor

**DOI:** 10.1002/advs.202516233

**Published:** 2025-10-24

**Authors:** Hongxia Li, Xiangyu Liu, Changshun Su, Xiaoguang Zhang, Chunyan Sun, Xu Yan

**Affiliations:** ^1^ Department of Food Quality and Safety College of Food Science and Engineering Jilin University Changchun 130062 P. R. China; ^2^ Key Laboratory of Advanced Gas Sensors College of Electronic Science and Engineering Jilin University Changchun 130012 P. R. China

**Keywords:** enzyme immobilization, hydrogen‐bonded organic framework, pesticide, plant‐wearable sensor

## Abstract

It is of great significance to on‐site detection of pesticides for food safety and pollution monitoring. Plant‐wearable sensors (PWS) are capable of real‐time detection of pesticides on crops, which facilitates the precise application of pesticides and determines whether the plant‐based foods meet the criteria for safe consumption. Herein, PWS are designed by embedding an acetylcholinesterase@poly(allylamine hydrochloride)‐hydrogen‐bonded organic frameworks (AChE@PAH‐HOF) in hydrogel discs to on‐site deliver pesticide‐residue information. The self‐assembly of AChE@PAH‐HOF, achieved via electronic interactions and hydrogen bonding with a crystalline structure‐directing agent, exhibits high catalytic activity and enhances stability. To facilitate long‐term and precision monitoring of organophosphorus pesticides, a PWS is constructed by integrating the AChE@PAH‐HOF into a glycerol‐sodium alginate hydrogel matrix, which provides high water‐retaining capacity essential for sustained operation under high‐temperature. By integrating with the image processing algorithm, the PWS successfully achieves in situ monitoring of pesticide degradation on tomato plants, demonstrating superior accuracy and sensitivity to chlorpyrifos with a detection limit of 1 ng mL^−1^. Such a PWS enables non‐invasive, cost‐effective pesticide detection in agricultural settings, supporting targeted crop health management and sustainable precision farming practices.

## Graphical abstract

By modulating the charge of acetylcholinesterase, it is encapsulated within HOF materials, thereby enhancing its stability and maintaining its activity. Subsequently, the composite material is embedded in a glycerol‐sodium alginate gel, achieving the long‐term monitoring of pesticides. Based on the capability of plant‐wearable sensor to provide accurate pesticide information, on‐site monitoring of chlorpyrifos degradation is realized on a tomato plant.

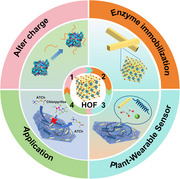

## 1 Introduction

Escalating global food demand, driven by demographic expansion on agricultural productivity, poses pressing challenges.^[^ ^1^, ^2^ ^]^ The agrochemical revolution enhanced crop yields and alleviated food scarcity, yet excessive pesticide use undermines food safety and induces chronic health risks through ecotoxicological accumulation.^[^ ^3^, ^4^, ^5^ ^]^ These concerns underscore the urgent need for advanced methodologies to precisely monitor agrochemicals (such as pesticides) in crops.^[^ ^6^, ^7^ ^]^ Conventional techniques, such as infrared thermography, hyperspectral imaging, and remote sensing,^[^ ^8^ ^]^ are limited by cost, complexity, and low temporal resolution, impeding in situ real‐time pesticide detection on plant surfaces. Recent significant advances in flexible sensing technologies have created new opportunities for plant communication.^[^ ^9^ ^]^ By transducing physiological and environmental cues into electrical or optical signals, enzyme‐based plant wearable sensors (PWS) have enabled dynamic surveillance of crop status with high precision.^[^ ^10^, ^11^ ^]^ Compared to invasive approaches, non‐invasive plant electrophysiology is advantageous in capturing genuine signals while minimizing damage to plant tissues. Despite their exceptional biorecognition and inherent signal amplification capabilities, enzymes in PWS exhibit low stability and poor catalytic activity under harsh on‐site conditions. This inherent limitation compromises the durability of the flexible substrates and, consequently, the overall stability and reliability of enzyme‐based PWS for agricultural applications.^[^ ^12^, ^13^ ^]^

Constraining native enzymes within engineered porous materials is an effective strategy for enhancing their biocatalytic stability.^[^ ^14^, ^15^ ^]^ Among these materials, hydrogen‐bonded organic frameworks (HOFs) are crystalline porous structures composed of organic building units assembled through intermolecular hydrogen bonds. Due to their mild synthesis conditions, gentle intermolecular interactions, and excellent pH stability, HOFs have emerged as promising candidates for enzyme immobilization.^[^ ^16^, ^17^ ^]^ In contrast, other commonly used porous frameworks present notable limitations. For instance, zeolitic imidazolate frameworks (ZIFs) are prone to structural degradation under mildly acidic conditions, and the narrow pore size (≈3.4 Å) limits accessibility to the immobilized enzyme.^[^ ^12^, ^18^, ^19^ ^]^ Covalent organic frameworks (COFs) typically rely on harsh reaction conditions (e.g., high‐temperature, high‐pressure, and high‐concentration acidic catalysts) to form stable covalent bonds, which may cause irreversible damage to the 3D structure of enzymes, leading to a significant decrease in their activity. In comparison, the mild preparation conditions and inherently flexible hydrogen‐bonded networks of HOFs help preserve the native conformation of enzymes, providing a favorable environment for maintaining high enzymatic activity.^[^ ^16^, ^20^, ^21^, ^22^ ^]^ However, existing HOF composites still face numerous challenges in enzyme immobilization applications. For instance, they generally exhibit low enzyme loading efficiency, and the narrow pore structure (≈6.4 Å) restricts the efficient transport of substrates and products, thereby affecting catalytic performance. Furthermore, the requirement for surface charge complementarity in current HOF immobilization platforms restricts their applicability to different types of enzymes to some extent. Therefore, developing an enzyme immobilization platform that combines high enzyme loading capacity, high catalytic activity, and good versatility holds significant research and practical value.

Compared to invasive approaches, non‐invasive plant electrophysiology is advantageous in capturing genuine signals while minimizing damage to plant tissues. The uneven and irregular surface topography of plants presents a big hurdle to intimately interface with the flexible substrate of PWS. Hydrogel substrates are crosslinked networks of hydrophilic polymer chains dispersed in water, which provide perspectives for the design and application of PWS.^[^ ^23^ ^]^ Along with these attributes, the softness, conformability, and biocompatibility of hydrogels have also laid the foundation for the development of PWS. However, hydrogels often suffer from insufficient adhesion and poor water retention, which can lead to decreased stability and fidelity of signal transmission, thereby limiting their long‐term application as sensors.^[^ ^24^, ^25^, ^26^ ^]^ Achieving long‐term, close contact between the sensor substrate and the plant surface without adversely affecting normal plant growth or sensor performance remains a critical challenge. Furthermore, despite successful monitoring of external leaf characteristics like temperature, humidity, and plant organ growth had been realized,^[^ ^27^, ^28^, ^29^, ^30^ ^]^ there is currently no PWS capable of noninvasive long‐term monitoring the pesticide residue information on individual plants. Therefore, creating such a PWS is both a significant challenge and an important goal with considerable practical value.

Herein, we designed a bio‐friendly facile approach to immobilize acetylcholinesterase (AChE) via electronic interactions and hydrogen bonding with a crystalline structure‐directing agent, which is employed as a model system to fabricate PWS. Structural analysis reveals the relevant assembly of AChE@poly(allylamine hydrochloride‐HOF(PAH‐HOF) via a hydrogen bond co‐assembly mechanism, obtaining insight into the interfacial interactions between HOFs and enzymes. Harnessing the superior catalytic bioactivity and outstanding stability, the AChE@PAH‐HOF was effectively employed as a recognition unit and signal amplifier to embed into glycerol‐sodium alginate hydrogel discs (G‐SA) for constructing PWS. Specifically, the immobilized AChE could catalyze the substrate to generate thiocholine (TCh), triggering a colorimetric reaction of 5,5′‐dithiobis‐(2‐nitrobenzoic acid) (DTNB). In the context of chlorpyrifos pesticide inhibition of the enzyme, the TCh‐driven signal transformation, coupled with an enzyme‐induced amplification protocol and an image processing algorithm, converts the pesticide concentration into digital information for the on‐site monitoring of pesticide residues. The G‐SA, offering high water‐retaining capacity essential for sustained operation under high‐temperature, enables the PWS to perform long‐term monitoring of pesticide levels. We accurately determined chlorpyrifos degradation in tomato plants by continuously capturing the color information of the PWS within 13 days, providing a reliable foundation for the advancement of precision agriculture and improving food safety and human health.

## 2 Results and Discussion

### 2.1 Structurally Characterizing AChE@PAH‐HOF Composites

**Figure 1:**
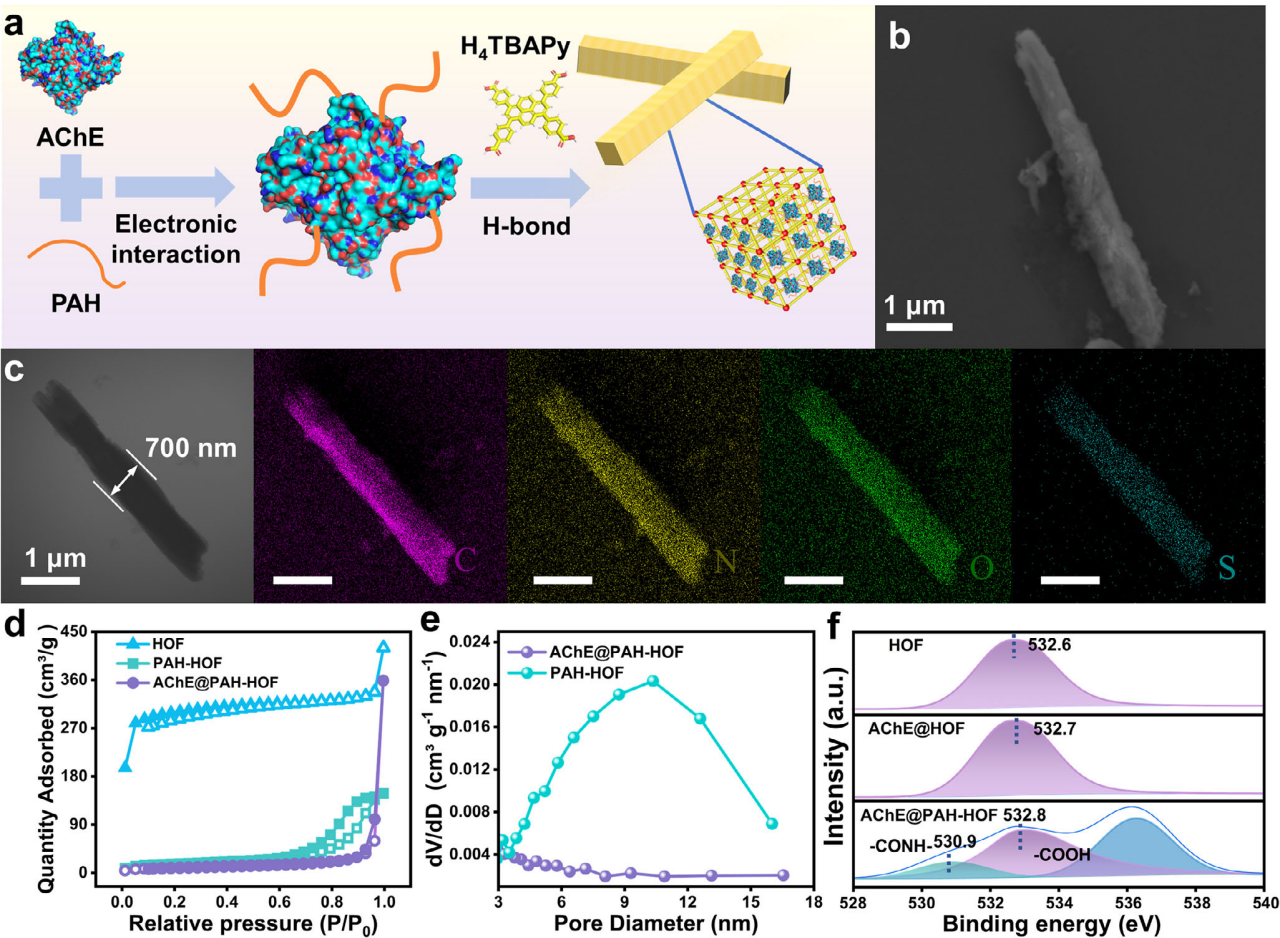
a) Process used to synthesize the AChE@PAH‐HOF composite. b) SEM images of AChE@PAH‐HOF. c) TEM images of AChE@PAH‐HOF. d) N_2_ adsorption/desorption isotherms of HOF, PAH‐HOF and AChE@PAH‐HOF. e) Pore size distribution of PAH‐HOF and AChE@PAH‐HOF. f) High‐resolution O1s XPS profiles of HOF, AChE@HOF and AChE@PAH‐HOF.

A PWS was designed by embedding AChE@PAH‐HOF (recognizing unit) into a water‐retaining hydrogel disc for monitoring of pesticides. The procedure of synthesizing AChE@PAH‐HOF is shown in **Figure** **1**a. A positively charged PAH was introduced to combine with the AChE via electrostatic interaction, followed by the introduction of 1,3,6,8‐Tetra(4‐carboxyphenyl)pyrene (H_4_TBAPy) as a structure directing agent to synthesize the AChE@PAH‐HOF through H‐bonds driven self‐assembly (Figure S1, Supporting Information). Scanning electron microscope (SEM) and Transmission electron microscopy (TEM) analyses revealed a rod‐like morphology with approximate dimensions of 4.0 µm in length and 0.7 µm in width (Figure 1b,c). Energy‐dispersive spectroscopy (EDS) elemental mapping confirmed the homogeneous distribution of C, N, O, and S (from AChE) elements throughout the entire architecture of AChE@PAH‐HOF (Figure 1c). The appearance of an amide I band (1659 cm^−1^) in Fourier‐transform infrared (FT‐IR) spectra of AChE@PAH‐HOF, characteristic of the enzyme skeleton, confirms the immobilization of AChE (Figure S2, Supporting Information).^[^ ^31^ ^]^ X‐ray photoelectron spectroscopy (XPS) showed the presence of the S element within AChE@PAH‐HOF also confirmed the embedding of the enzyme (Figure S3, Supporting Information). X‐ray diffractometry (XRD) revealed that AChE@PAH‐HOF has a similar pattern to that of pure PAH‐HOF, confirmings that the crystalline structure of PAH‐HOF is preserved after AChE incorporation (Figure S4, Supporting Information). To closely examine protein dispersibility within the PAH‐HOF composite, we employed protein‐entrapped gold nanoclusters (AuNCs) as a structural probe to synthesize protein@PAH‐HOF. TEM image coupled with EDS mapping confirmed the uniform dispersion of AuNCs throughout the intact PAH‐HOF structure (Figure S5, Supporting Information). Brunauer–Emmett–Teller (BET) analysis and Barrett–Joyner–Halenda (BJH) pore‐size distribution data indicated that AChE coating slightly reduced both surface area and average pore diameter (Figure 1d,e). Such a reduction of pore diameter was caused by embedding AChE to occupy the aperture space.

Comparative TEM analysis of AChE@PAH‐HOF composite, AChE@HOF composite, and pure HOF revealed that the incorporation of the enzyme induced distinct morphological changes in the HOF structure. The immobilization of AChE significantly increased the average HOF width from 240 to 500 nm (Figures S6–S8, Supporting Information). The subsequent incorporation of PAH further expanded the width to 750 nm (Figure 1c; Figures S7 and S8, Supporting Information). This morphological change likely stems from the influence of enzyme charges and amino acid residues on HOF assembly. High‐resolution XPS analysis revealed that O1s signals originated primarily from amide oxygen (─CONH─, green) and carboxyl oxygen (─COOH, purple). While pure HOF, AChE@HOF composite, and AChE@PAH‐HOF composite exhibited strong ─COOH peaks, a distinct ─CONH─ peak was uniquely observed in AChE@PAH‐HOF (Figure 1f), indicating that PAH introduces additional binding sites via amide linkages between the HOF ligand and the AChE‐PAH complex.

**Figure 2:**
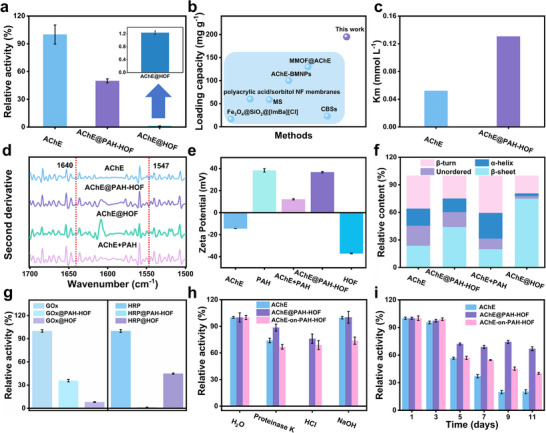
a) The relative activity of AChE, AChE@PAH‐HOF, and AChE@HOF. b) The comparison of different loading methods. c) The Km of AChE@PAH‐HOF and AChE. d) Second derivative FT‐IR spectra. e) The zeta potential of AChE, PAH, AChE+PAH, AChE@PAH‐HOF, and HOF. f) Secondary structure contents of AChE, AChE@PAH‐HOF, AChE + PAH, and AChE@HOF. g) The relative activity of GOx, GOx@PAH‐HOF, GOx@HOF, HRP, HRP@PAH‐HOF and HRP@HOF. h) Stability of AChE@PAH‐HOF, AChE, and AChE‐on‐PAH‐HOF of proteinase K (0.1 mg mL^−1^), HCl (1 mm), and NaOH (1 mm). i) Long‐term stability of AChE, AChE@PAH‐HOF, and AChE‐on‐PAH‐HOF.

To elucidate the mechanistic impacts of the introduction of PAH, the catalytic activity of AChE@PAH‐HOF was investigated using a chromogenic biochemical reaction of acetylthiocholine iodide (ATCh) and DTNB.^[^ ^32^ ^]^ Notably, AChE@HOF possesses low activity while AChE@PAH‐HOF is capable of catalyzing ATCh with high activity (41.5 times higher than AChE@HOF) (**Figure** **2**a). AChE@PAH‐HOF exhibited a high activity recovery of 49.9% with a quantitative enzyme loading efficiency of 195 mg g^−1^ (Figure 2b). Comparative studies with other representative frameworks demonstrated that PAH‐HOF achieved markedly superior encapsulation efficiency (58.5%, 1.95–10.6‐fold higher than others) along with enhanced relative activity (Figures S9–S11; Table S1, Supporting Information), thereby demonstrating its distinct advantage as a framework for enzyme encapsulation.^[^ ^33^ ^]^ Circular dichroism spectroscopy (CD) showed that the spectral peak positions are consistent when PAH is combined with the enzyme, confirming that PAH has little influence to the protein structure (Figure S12, Supporting Information). Thus, the PAH‐induced modification selectively alters the surface charge distribution of AChE while preserving its native conformational integrity. Ultraviolet spectroscopy (UV) and fluorescence spectroscopy analyses of the enzyme structure in the absence and presence of PAH were investigated. The spectral peak positions after the addition of PAH remain essentially consistent compared to those of free AChE. Such observation is supported by a similar phenomenon observed with other enzymes (Figures S13 and S14, Supporting Information). This effect likely arises from the dominant non‐covalent PAH‐enzyme interactions and PAH's substantial molecular size (17.5 kDa). The resulting steric hindrance prevents PAH penetration into the enzyme interior, excluding binding at the active site while leaving the enzyme structure unperturbed.

Further analysis of enzymatic kinetics revealed that the Michaelis–Menten constant of AChE@PAH‐HOF (Km = 0.1305 mmol L^−1^) was 2‐fold higher than that of free AChE (Km = 0.052 mmol L^−1^), indicating that the substrate affinity was slightly reduced upon encapsulation (Figure 2c; Figure S15, Supporting Information).^[^ ^34^ ^]^ The Vmax of AChE@PAH‐HOF (0.1864 nM L^−1^ min^−1^) was lower than that of free AChE (Vmax = 1.263 nM L^−1^ min^−1^). This result may be attributed to the slight blockage of the substrate diffusion, surface‐crowding effect, and enzyme orientation‐caused steric hindrance of the HOF. Such a phenomenon is present in most of the fixed materials (MOFs, COFs, and HOFs). Second‐derivative FT‐IR spectra revealed strong absorption of AChE at the amide I (1640 cm^−1^) and amide II (1547 cm^−1^) bands. Compared to other composites, only the diffraction peaks of AChE@PAH‐HOF and AChE+PAH remained unchanged (Figure 2d; Figure S16, Supporting Information), these results indicate that PAH‐HOF effectively maintains the native conformation of the enzyme. The zeta potential measurements of AChE@PAH‐HOF components revealed that PAH carries a positive charge while AChE is negatively charged (Figure 2e). Upon complex formation, the resultant composite adopts a net positive charge, facilitating rapid nucleation. These may be a key factor in maintaining the enzymatic activity during immobilization.^[^ ^18^ ^]^ Gaussian multicomponent fitting was employed to evaluate alterations in the secondary structure of the composites. The similar secondary structure between free AChE and AChE@PAH‐HOF indicated that the enzyme's structural integrity was largely preserved after immobilization. In contrast, a noticeable change in α‐helix content and β‐sheet was observed after the immobilization of the other framework (Figure 2f; Figure S17, Supporting Information). This alteration may be attributed to the structural disruption during the immobilization process. These results revealed that while HOF immobilization induced structural deformation of the enzyme, the introduction of PAH effectively maintained its native conformation through protective molecular interactions, demonstrating a crucial role in stabilizing the biocatalytic architecture during the assembly process of AChE@PAH‐HOF.

To investigate the universality of the strategy, experiments using enzymes with different charges were carried out to compare the activity after immobilization, including negatively charged glucose oxidase (GOx) and positively charged horseradish peroxidase (HRP), cytochrome C (Cyt C).^[^ ^18^ ^]^ GOx@PAH‐HOF displayed a 4.46‐fold enhancement in activity over GOx@HOF, whereas the activities of HRP@PAH‐HOF and Cyt C@PAH‐HOF were significantly lower than those of the corresponding HOF composites. (Figure 2g; Figure S18, Supporting Information). Furthermore, the apparent Michaelis constant (Km) of the HRP@PAH‐HOF composite increased slightly relative to free HRP, indicating reduced substrate affinity. In contrast, GOx@PAH‐HOF maintained a Km value comparable to free GOx, preserving its inherent substrate affinity (Figures S19 and S20, Supporting Information). We also compared the loading efficiency of different enzymes (Figure S21, Supporting Information) and found that positively charged enzymes exhibited lower loading efficiency. This can be attributed to competitive interactions between PAH and the positively charged enzyme during immobilization, which hindered the incorporation of PAH into the positively charged particles and consequently diminished both loading efficiency and catalytic activity. In contrast, when the negatively charged enzyme reacts with PAH through electrostatic adsorption, the enzyme/PAH complex interacts with the ligand H_4_TBAPy to form enzyme@PAH‐HOF, offering a valuable reference for the subsequent encapsulation of the enzyme.

To assess the protective properties of HOF nanostructure, free AChE, AChE@PAH‐HOF composite, and AChE‐on‐PAH‐HOF (AChE linked on the surface of PAH‐HOF) composite were treated with hydrochloric acid, proteinase K, and sodium hydroxide. Notably, after 40 min of treatment with 1 mm hydrochloric acid, the activity of free AChE is essentially inactivated (decreased to 1%), whereas AChE@PAH‐HOF retained 88% activity (Figure 2h). Encapsulation within AChE@PAH‐HOF enhanced AChE's tolerance to sodium hydroxide (1 mm), maintaining over 80% activity. We also compared the tolerance of AChE, AChE@PAH‐HOF, and AChE‐on‐PAH‐HOF to protein hydrolyses. After treatment with trypsin (0.1 mg mL^−1^) for 24 h, the AChE showed a ≈90% loss of activity, whereas AChE@PAH‐HOF retained 98.1% activity, demonstrating the protective effect of HOF on the embedded enzyme (Figure S22, Supporting Information). The recyclability tests and long‐term stability of AChE@PAH‐HOF demonstrate the retention of enzymatic activity exceeding 80% through 6 cycles and maintaining above 70% over 11 days. (Figure 2i; Figure S23, Supporting Information). All results demonstrated that AChE@PAH‐HOF enhances the stability and recyclability of AChE.

### 2.2 Evaluating the Performance of AChE@PAH‐HOF Hydrogels

**Figure 3:**
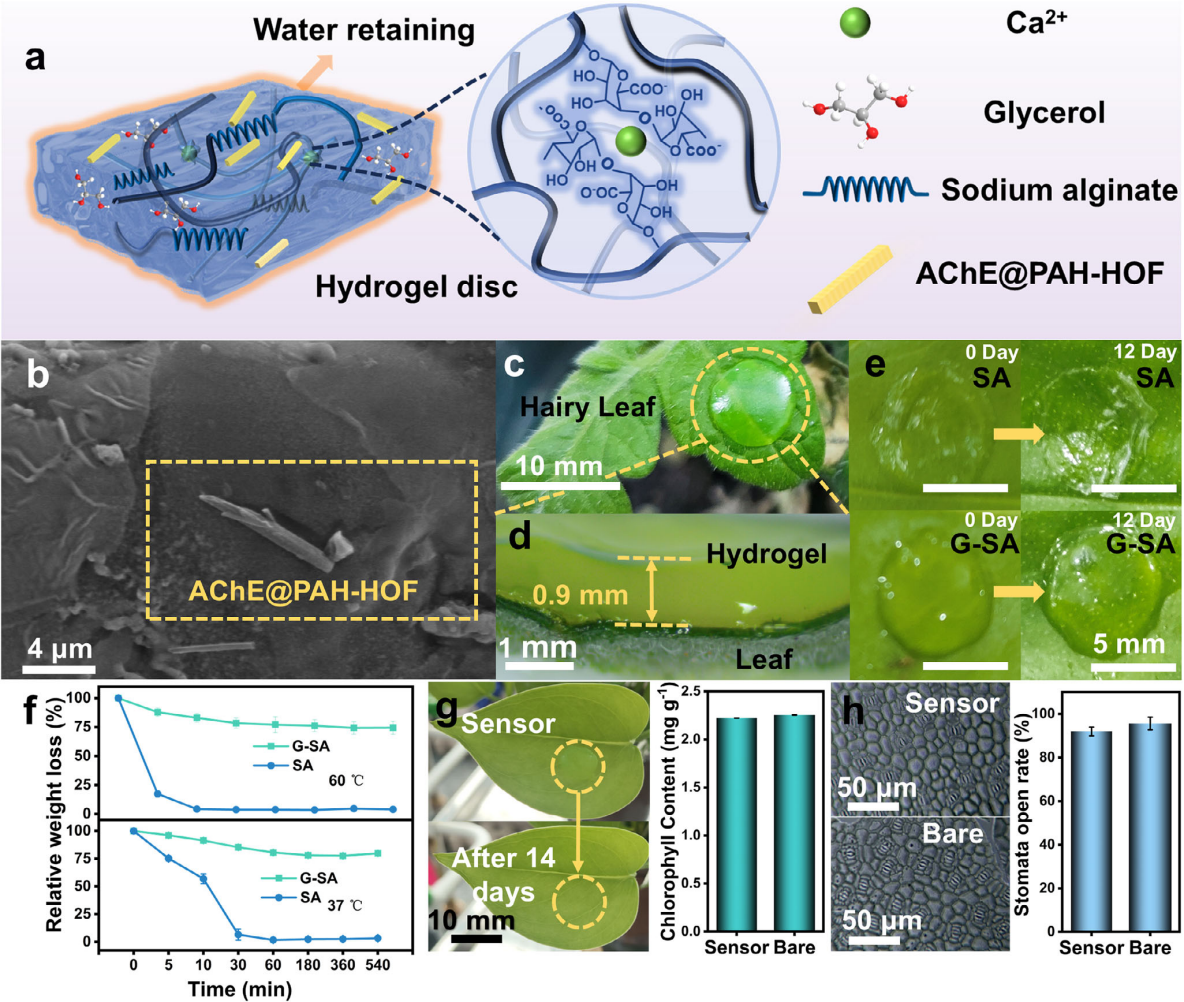
a) The preparation of PWS. b) The SEM image of a PWS. c) Optical photographs of the PWS on a plant leaf. d) Cross‐sectional contact interface between PWS and leaf blade. e) The morphological changes of hydrogels stored at room temperature for 12 days. f) The weight retention behavior of SA and G‐SA under different temperatures (37 and 60 °C). g) The changes in chlorophyll after sensor attachment to plants. h) Stomatal morphology on leaf surfaces and comparison of opening ratios between bare and sensor‐attached areas.

The hydrogel‐based PWS was fabricated by immobilizing AChE@PAH‐HOF composites via diffusion within a gel solution (**Figure** **3**a). This system leverages Ca(II)‐mediated self‐assembly of sodium alginate and glycerol‐triggered water‐retaining capacity, generating superior intermolecular cooperativity and enhanced cross‐linking strength.^[^ ^35^ ^]^ The G‐SA, formed in a 3.5 mm radius × 0.9 mm thickness mold, exhibits robust cross‐linked networks. The analysis of the PWS's microstructure demonstrated the successful integration of AChE@PAH‐HOF within the G‐SA (Figure 3b; Figure s24, Supporting Information).

The PWS sensors demand operational robustness against mechanical deformation and extreme‐field environments. Figure 3c shows the PWS mounted on a tomato leaf, demonstrating its lightweight design that avoids mechanical stress on plant tissues, confirming its suitability for real‐time in situ applications. This was further validated by the optical image of the PWS attached to the tomato stem (Figure s25, Supporting Information). Optical microscopy and SEM imaging of PWS cross‐sections, combined with rheological characterization, demonstrated that the PWS exhibit exceptional flexibility and enable conformal bioadhesion to plant leaf surfaces (Figure 3d; Figures S26 and S27, Supporting Information). External factors, including temperature, humidity, and airflow, can cause significant changes in the water‐retaining capacity and functionality of the hydrogel sensor,^[^ ^36^ ^]^ potentially affecting its sensing performance during testing. To minimize the impact of water evaporation, glycerol was incorporated into the hydrogel discs. The trihydroxy architecture of glycerol establishes hydrogen‐bonding interactions with free water molecules, effectively immobilizing mobile water phases through molecular confinement.^[^ ^37^ ^]^ The measured water contact angle confirms the G‐SA's hydrophilic nature (Figure S28, Supporting Information). The water retention capacity of the hydrogel was evaluated, revealing a substantial enhancement with increasing glycerol content. Among the evaluated formulations, the hydrogel containing 60% glycerol exhibited the highest water retention capacity (Figure S29, Supporting Information). Following 96‐h thermal exposure at 37 °C, the 60% glycerol‐incorporated hydrogel exhibited 67% mass retention, in stark contrast to the non‐glycerol counterpart, which demonstrated severe mass depletion (3.85% residual mass). When hydrogel discs adhered to plant surfaces were maintained at room temperature (25 °C) for 12 days, G‐SA retained their structural integrity without significant morphological changes, whereas sodium alginate hydrogel discs (SA) exhibited substantial structural collapse (Figure 3e; Figure S30, Supporting Information). To further validate this property, the capacity of water retention of G‐SA was monitored at 25 °C, 37 °C, 45 °C, and 60 °C (Figure 3f; Figure S31, Supporting Information). The swelling ratio and evaporation rate of the samples were measured under different environmental conditions. G‐SA showed reduced swelling and slower evaporation relative to SA, highlighting its superior water‐retention capability (Figures S32–S34, Supporting Information). Tensile data also show that the glycerol‐incorporated gel has superior mechanical properties and remains stable after equilibration at high‐temperatures and humidity levels. In contrast, the conventional gel rapidly dries and becomes increasingly brittle under elevated temperatures (Figure S35, Supporting Information). The results demonstrated that glycerol markedly improved the hydrogel's ability to retain water under varying thermal conditions.

A high‐performance plant‐compatible sensor possesses both superior mechanical characteristics and negligible impact on plant functions during application. To evaluate PWS effects on leaf growth, chlorophyll content changes between treated leaves and untreated leaves were monitored (Figure 3g). Comparative analysis demonstrated minimal physiological impact from sensor attachment, with chlorophyll content in sensor‐equipped leaves measuring 2.22 vs 2.26 mg g^−1^ in untreated controls. The results demonstrated that the sensor did not affect chlorophyll content in leaves for 14 days. As respiration is crucial for plant growth, we examined the state of the stomata, which are regulatory pores that mediate gas exchange between the dry atmosphere and the hydrated substomatal cavities.^[^ ^38^ ^]^ After 2 h of the PWS installation on the abaxial leaf surface, stomatal morphology showed no structural alterations (Figure 3h), suggesting no significant interference with stomatal‐mediated respiration. These findings demonstrate the biocompatibility of PWS in terms of chlorophyll metabolism and stomatal function, supporting their potential as a minimally invasive plant sensor interface. The comprehensive experimental data collectively validate the exceptional biocompatibility of the PWS.

**Figure 4:**
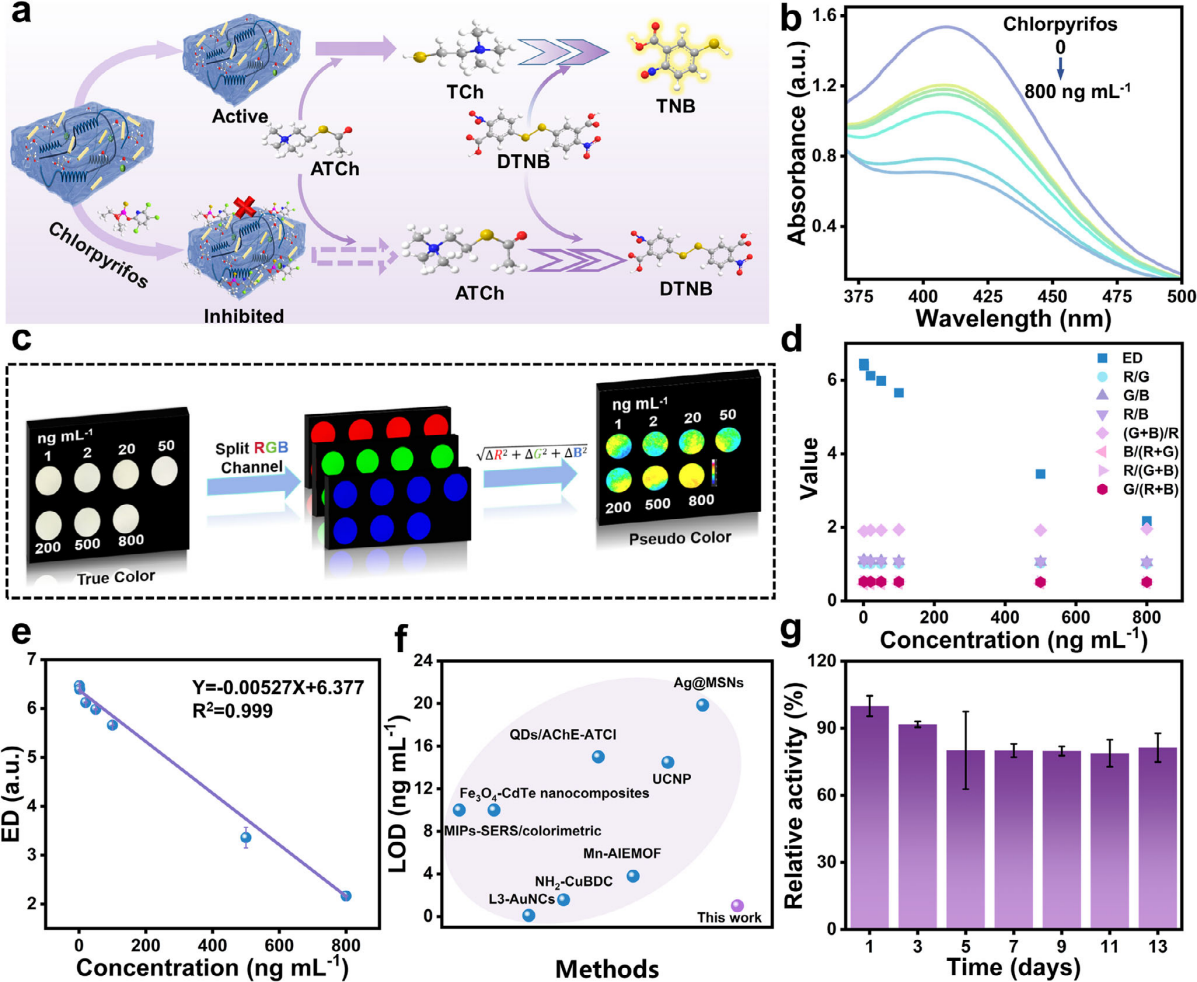
a) Schematic depicting the chlorpyrifos detection principle. b) UV spectra acquired in the presence of increasing concentrations of chlorpyrifos (0–800 ng mL^−1^). c) True‐color images are split into RGB channels, and the value response is calculated to obtain pseudo color. d) Superiority of ED compared with other color‐processing algorithms. e) Relationship between ED response and chlorpyrifos concentration. f) Comparative analysis of the detection limits for chlorpyrifos determination. g) The long‐term stability of the PWS.

Functioning as an interface linking plants and biosensing systems, AChE@PAH‐HOF composite catalyzes the hydrolysis of ATCh to generate TCh, which could react with DTNB to produce a yellow‐colored 5‐thio‐2‐nitrobenzoic acid (TNB) (**Figure** **4**a). Chlorpyrifos (an organophosphate pesticide) inhibits AChE activity, reducing TCh production and thereby attenuating the yellow color development in the PWS. Consequently, chlorpyrifos indirectly regulates the colorimetric intensity of PWS in a concentration‐dependent manner with high reproducibility (Figure 4b). The results show that the color of the PWS becomes progressively light as the concentration of chlorpyrifos increases (Figure 4c). Aiming for on‐site monitoring with a readout signal, we collected concentration‐dependent photo information instead of spectra acquired by large‐scale imaging systems or optical/electronic apparatus. The image processing algorithm separated the projected chromogenic signals into red (R), green (G), and blue (B) channels. The color response (Δ*R*, Δ*G*, and Δ*B*) of the PWS to chlorpyrifos exposure was then calculated by subtracting the pre‐exposure control image from the post‐exposure image, enabling quantitative determination of the Euclidean distance (ED) (Figure 4c). The ED quantifies the linear displacement between two points within the RGB color space, effectively capturing the chromatic variations that were observed, which demonstrates superior results compared to other color‐processing algorithms (Figure 4d). To facilitate visual interpretation, the colorimetric responses were normalized and translated into a pseudo‐color scale, enabling the construction of a differential color map that accentuates changes in pesticide concentration. Based on a pseudo‐color algorithm, the hydrogel sensors have a great linear equation (Y = −0.0052X + 6.377) with a linear range (1.0–800 ng mL^−1^) (Figure 4e). The limit of detection (LOD) was determined to be 1.0 ng mL^−1^, demonstrating higher sensitivity compared to reported strategies (Figure 4f). Through the combination of colorimetric reactions and image processing software, the concentration of pesticides can be detected without the need for complex and large‐scale instruments. Stored under controlled conditions, the sensor maintained stable performance over 13 days (Figure 4g), indicating that PWS is suitable for long‐term surveillance of pesticide residues.

### 2.3 On‐Site Monitoring of Pesticide Degradation

**Figure 5:**
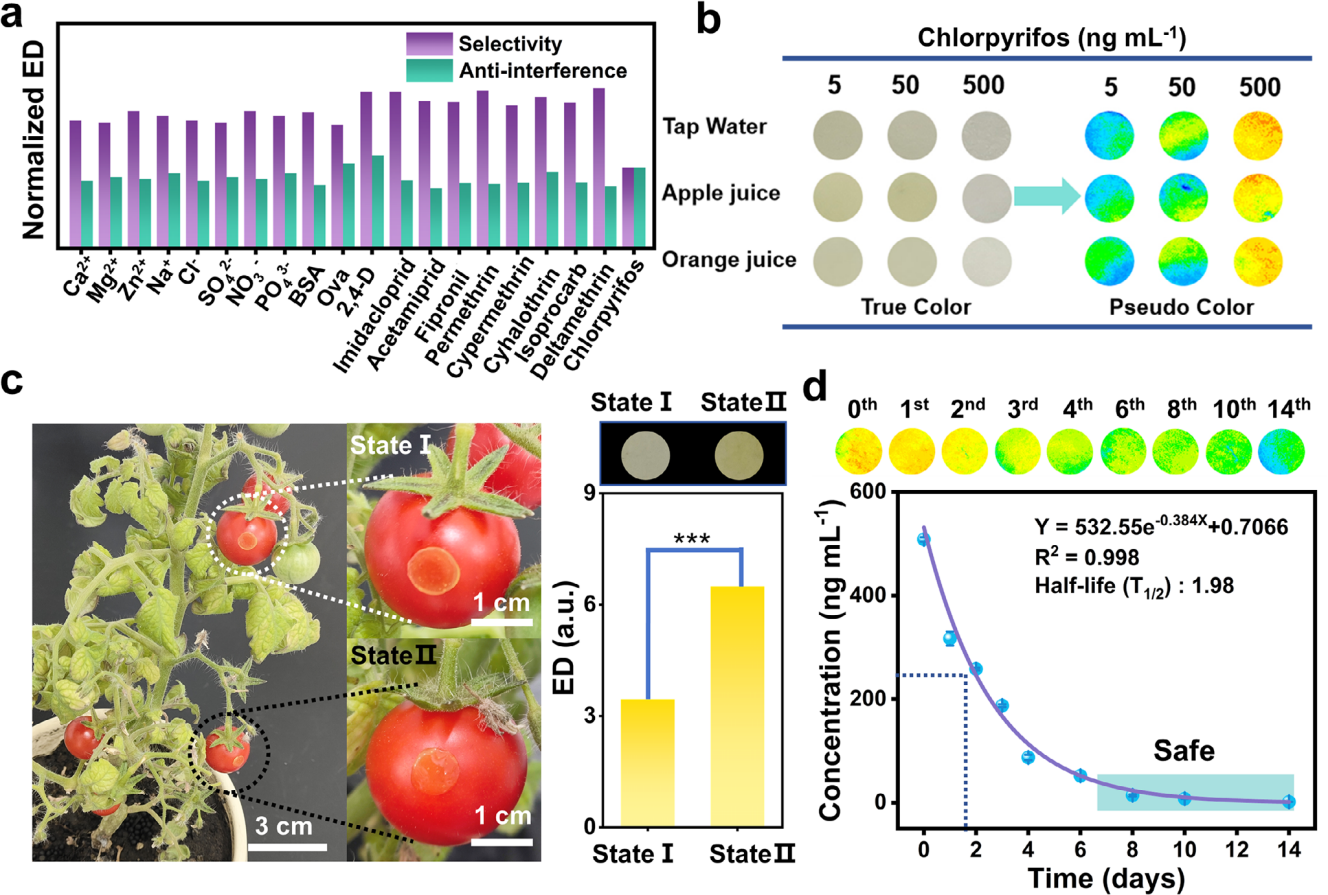
Practical applications of PWS. a) Selectivity and anti‐interference of PWS. b) Image of the PWS with spiked tap water, apple juice and orange juice. c) Responses of PWS to the fruit of tomato in the presence and absence of chlorpyrifos. d) Chlorpyrifos degradation on leaves over 14 days.

To evaluate the selectivity and anti‐interference capability of the PWS, experimental assessments were conducted by adding various ions, proteins, and other potential interfering substances. This approach effectively simulates the complex chemical environment encountered in practical applications, thereby verifying the stability and selectivity of the sensor in the presence of multiple interfering factors. The presence of interferents resulted in negligible changes in the ED signal, even at concentrations 10 times higher than that of chlorpyrifos. The data demonstrate that coexisting compounds have no negative effect on the recognition capability of the hydrogel discs (**Figure** **5**a; Figures S36 and S37, Supporting Information). The outstanding selectivity and anti‐interference capability likely stem from the combined sieving effects of AChE@PAH‐HOF, the hydrogel disc network, and the specific recognition of AChE. This synergy restricts the entry of substances based on size and enables selective binding to chlorpyrifos. We further tested the reliability of the PWS by spiking a certain amount of pesticide into tap water, apple juice, and orange juice samples (Figure 5b). The addition of chlorpyrifos to spiked samples demonstrated a high inhibitory capacity against the activity of AChE@PAH‐HOF, with recoveries ranging from 96.2% to 108.0% and a relative standard deviation within 9.72% (Table S2, Supporting Information). Such pesticide recoveries of PWS were further validated by High‐Performance Liquid Chromatography (HPLC), which exhibited a significant correlation (the average difference between the two assays is 0.02148) with an established portable biosensor, thereby confirming accuracy (Figure S38, Supporting Information).

Understanding the degradation of pesticides provides strong guidance for the agricultural application of pesticides. To evaluate the practicality of the PWS, we sprayed chlorpyrifos pesticide on tomato plants and monitored the pesticide degradation. Hydrogel discs were positioned at varied locations to mitigate measurement errors arising from sample‐to‐sample variability. This systematic sampling approach effectively minimized the spatial bias typically associated with single‐point detection. As demonstrated in Figure 5c, PWS enabled spatiotemporal monitoring of chlorpyrifos degradation across tomato plant leaves and temporal phases. The PWS demonstrated a concentration‐dependent chromatic response, exhibiting distinct yellow coloration in pesticide‐free conditions that gradually faded with increasing chlorpyrifos concentration. The results demonstrate that the concentration of chlorpyrifos is relatively high at the initial time point (505.8 ng mL^−1^) and gradually decreases over time. The degradation follows a pseudo‐first‐order reaction kinetics model (Y = 532.55 e^−0.864X^ + 0.7066) with a half‐life (T_1/2_) of 1.97 days (Figure 5d). The pesticide content tracking experiment indicates that after the application of chlorpyrifos, the pesticide concentration enters a safe level within 6 to 8 days (safety interval). Therefore, the PWS successfully achieved in situ detection of chlorpyrifos degradation in the plant. This PWS delivers precise detection capabilities through non‐invasive operation, preserving system integrity while boosting monitoring efficiency. It significantly minimizes operational complexity and eliminates the high costs inherent in traditional detection methods. By analyzing dynamic changes in pesticide residues, this technology helps determine whether agricultural products meet safety standards for harvesting, providing significant support for precision agriculture and food safety regulation.

## 3 Conclusion

In summary, a highly robust PWS was innovatively designed and developed for monitoring pesticide degradation based on AChE@PAH‐HOF composite and high water‐retaining hydrogel discs. The self‐assembly of AChE@PAH‐HOF, driven by electronic interactions and hydrogen bonding with a crystalline structure‐directing agent, demonstrated a high protein encapsulation efficiency (58.5%) and significantly enhanced stability. Structural analysis reveals the relevant assembly of AChE@PAH‐HOF via a hydrogen bond co‐assembly mechanism, obtaining insight into the interfacial interactions between HOFs and enzymes. Harnessing the superior catalytic bioactivity and outstanding stability, the AChE@PAH‐HOF was effectively employed as a recognition unit and signal amplifier to embed into G‐SA for constructing PWS. The PWS exhibited exceptional environmental adaptability and high water‐retaining capacity, maintaining great morphology under room temperature after 12 days, while achieving bioadhesion to a heterogeneous plant surface. Critically, the PWS showed full compatibility with plant physiology, inducing no measurable perturbations in respiration or chlorophyll content. Functioning as a colorimetric element, the PWS achieves reaction system miniaturization while simultaneously enabling pesticide residue quantification, effectively simplifying experimental workflows. The PWS enables in situ monitoring of chlorpyrifos residues across plant surfaces with a detection limit of 1 ng mL^−1^, allowing real‐time mapping of pesticide degradation through direct attachment to plant organs. PWS provides an accurate and rapid detection method of chlorpyrifos, addressing food safety requirements. We envisage that the AChE@PAH‐HOF‐based PWS is anticipated to catalyze advancements in intelligent agricultural technologies.

## 4 Experimental Section

### Synthesis of AChE@PAH‐HOF

The synthesis method was modified from the previously reported literature.^[^ ^21^ ^]^ Typically, Poly(allylamine hydrochloride) (PAH, 4 mg) was dissolved in 4 mL of Tris‐HCl (50 mm, pH = 8). Acetylcholinesterase (AChE, 4 mg) was dissolved in 4 mL of Tris‐HCl (50 mm, pH = 8). The mixture was stirred at 4 °C for 5 min, and then 1,3,6,8‐Tetra(4‐carboxyphenyl) pyrene (H_4_TBAPy, 8 mg) was dissolved in N, N‐Dimethylformamide (DMF, 1 mL). The mixture was stirred at 4 °C for 5 min, and then aged for 10 min at 4 °C. Subsequently obtaining the yellow precipitates (Enzyme@PAH‐HOF) were obtained. Enzyme@PAH‐HOF was washed three times with Tris‐HCl buffer (50 mm, pH = 8.0) and recovered by centrifugation (12 000 g, 10 min).

### Preparation of AChE@PAH‐HOF‐Based Hydrogels

Briefly, 2.5 mL of AChE@PAH‐HOF (2.0 mg mL^−1^) solution was mixed with 2.5 mL of glycerol‐sodium alginate (40 mg mL^−1^). Then, 25 µL of the above‐mentioned mixture was added to a mold. And the calcium chloride (0.1 m) dissolved in a 60 percent glycerol aqueous solution was dripped on the mold. The hydrogel was formed at room temperature for several minutes and stored at 4 °C for use later.

### Determination of Chlorpyrifos by PWS

Chlorpyrifos solution with different concentrations (25 µL) and the AChE@PAH‐HOF hydrogel were evenly mixed in a 1.5 mL microtube and incubated at 37 °C for 40 min. Then, 10 mm ATCh (25 µL) and 0.3 mg mL^−1^ DTNB (25 µL) were added for incubating another 30 min. The color images were captured via smartphone camera and analyzed with ImageJ software. Additional experimental details are provided in the Supporting Information.

## Conflict of Interest

The authors declare no conflict of interest.

## Supporting information



Supporting Information

## Data Availability

The data that support the findings of this study are available from the corresponding author upon reasonable request.
