# Supplementary material for: Enzyme Immobilization by Hydrogen‐Bonded Organic Framework for the Construction of Plant‐Wearable Sensor
Source: Adv Sci (Weinh). 2025 Oct 24;13(3):e16233. doi: 10.1002/advs.202516233 (PMC12806410; doi:10.1002/advs.202516233)
Supplement: Supplementary file 1 — Supporting Information [file ADVS-13-e16233-s001.docx]

**Supporting Information**

**Enzyme Immobilization by Hydrogen-Bonded Organic Framework for the Construction of Plant-Wearable Sensor**

Hongxia Li^a^, Xiangyu Liu^a^, Changshun Su^a^, Xiaoguang Zhang^a,*^, Chunyan Sun^a,*^, Xu Yan^b,*^

^a^ Department of Food Quality and Safety, College of Food Science and Engineering, Jilin University, Changchun 130062, P. R. China

^b^ Key Laboratory of Advanced Gas Sensors, College of Electronic Science and Engineering, Jilin University, Changchun 130012, P. R. China

^*^ Corresponding author

Email: [yanx@jlu.edu.cn](mailto:hxiali@jlu.edu.cn)

sunchuny@jlu.edu.cn

xiaoguang61@jlu.edu.cn

**1. Experimental Sections**

**1.1 Materials**

1,3,6,8-Tetra(4-carboxyphenyl)pyrene (H_4_TBAPy), poly(allylamine hydrochloride) (PAH), acetylthiocholine (ATCh), glucose oxidase (GOx), peroxidase from horseradish (HRP), acetylcholinesterase (AChE), 5,5'-Dithiobis-(2-nitrobenzoic acid) (DTNB), 3,3′,5,5′- tetramethylbenzidine (TMB), N,N-Dimethylformamide (DMF), Coomassie Brilliant Blue G-250, Dimethyl sulfoxide (DMSO), sodium alginate, glycerol, trypsin, and Ova Peptide were all purchased from Shanghai Macklin Biochemical Technology Co., Ltd. China). Gold chloride trihydrate was purchased from Shanghai Aladdin Biochemical Technology Co., Ltd. Bovine serum albumin, fraction V, heat shock, Cytochrome C (Cyt C), Tris-HCl (1 M, pH=8), isolation, zinc nitrate, trisodium phosphate, calcium chloride, and magnesium sulfate were all purchased from Sangon Biotech (Shanghai) Co., Ltd. Phosphoric acid was purchased from Beijing Institute of Chemical Reagents Co., Ltd Hydrogen peroxide (H_2_O_2_) was purchased from Sinopharm Chemical Reagent Co., Ltd. Pesticides are bought from Tianjin Zhongyi Jiaxin Technology Development Co., Ltd. Methanol was purchased from Shanghai Anpel Experimental Technology Co., Ltd.

**1.2 Characterization**

The microstructures are recorded using JEM-2100 transmission electron microscope (TEM) and JEM-7500 scanning electron microscope (SEM). X-ray photoelectron spectroscopy (XPS) measurement is performed using an American Thermo Fisher 250XI photoelectron spectrometer. The phase and crystalline information of the samples is examined by XRD (Rigaku D/MAX-2550, Cu-Kα, λ = 1.5418 Å). The absorption spectrum was measured by Shimadzu UV-270001 ultraviolet-visible spectrophotometer. We used an RF-5301 PC spectro fluorophotometer (Shimadzu, Japan) to acquire FL spectra. Fourier Transform Infrared (FT-IR) spectra were recorded on a Shimadzu IRPrestige-21 spectrometer. The Circular Dichroism spectroscopy was measured by PMS 450. Zeta potential measurements were performed on a Zetasizer-Nano ZSZEN3600 apparatus (Malvern Instruments, UK).

**2. Methods**

**2.1 Preparation of Enzyme@PAH-HOF**

The synthesis method was modified from the previously reported literature^[1]^. Typically, poly(allylamine hydrochloride) （PAH, 4 mg） was dissolved in 4mL of Tris-HCl (50 mM, pH=8). Enzyme (acetylcholinesterase (AChE,4 mg), glucose oxidase (GOx, 4 mg), heroxidase from horseradish (HRP, 4 mg), Cytochrome C (Cyt C)) was dissolved in 4mL of Tris-HCl (50 mM, pH=8). The mixture was stirred at 4 ℃ for 5 min, and then adding 1,3,6,8-Tetra(4-carboxyphenyl)pyrene (H_4_TBAPy, 8 mg) was dissolved in N,N-Dimethylformamide (DMF, 1 mL). And then aged for 10min at 4 ℃. Subsequently obtaining the yellow precipitates (Enzyme@PAH-HOF). Enzyme@PAH-HOF was washed three times with Tris-HCl buffer (50 mM, pH 8.0) and recovered by centrifugation (12,000 g, 10 min).

**2.2 Preparation of Enzyme@HOF**

The synthesis method was modified from the previously reported literature^[2]^. Typically, Enzyme (5 mg) was dissolved in 8 mL of deionized water. The H_4_TBAPy (10 mg) was dissolved in DMF (10 mL), then, the mixture was stirred at room temperature for 10 min. Subsequently obtain the yellow solution. Enzyme@HOF was washed three times with deionized water and recovered by centrifugation (10,000 rpm, 10 min).

**2.3 Preparation of HOF**

150 mg H_4_TBAPy (0.225 mmol) was dissolved in 22.5 mL of DMF to which 90 mL MeOH was added and stirred for 1minutes. The mixture was stood at room temperature for 12 hours to afford yellow block crystals of HOF. Finally, the products were collected by centrifugation (10,000 rpm, 10 min), washed by ethanol for three times and dried under vacuum at room temperature.

**2.4 Preparation of AChE-on-PAH-HOF**

Typically, PAH (4 mg) was dissolved in 4mL of Tris-HCl (50 mM, pH=8) and mixed with H_4_TBAPy (8 mg) which was dissolved in DMF (1 mL). The mixture was stirred at 4 ℃ for 10 min. And then, adding AChE (4 mg) was dissolved in 4mL of Tris-HCl (50 mM, pH=8). The mixture was aged for 5min at 4 ℃. Subsequently obtaining the yellow precipitates. AChE-on-PAH-HOF was washed three times with Tris-HCl buffer (50 mM, pH=8.0) and recovered by centrifugation (12,000 g, 10 min).

**2.5 Preparation of AChE@PAH-HOF-based hydrogels.**

Briefly, 2.5 mL of AChE@PAH-HOF (2.0 mg mL^-1^) was mixed with 2.5 mL of glycerol-sodium alginate (20 mg mL^-1^). Then, 25 μL of the above-mentioned mixture was added into a mold. And the calcium chloride (0.1 M) dissolved in 60 percent glycerol aqueous solution was dripped on the mold. The hydrogel was formed at room temperature for several minutes and stored at 4 °C for later use.

**2.6 Construction of Brandford Standard Curve**

The Bradford reagent was prepared by dissolving 5 mg Coomassie Brilliant Blue G-250 in a solvent mixture containing 2.5 mL ethanol, 5 mL phosphoric acid, and 42.5 mL deionized water. Thus, we prepared AChE solutions at varying concentrations (0.1, 0.2, 0.3, 0.4, 0.5, 0.6, 0.8, and 1.0 mg mL^-1^). Aliquoting (20 μL) of each solution were dispensed into microplate wells, followed by addition of 200 μL Brandford Solution. After incubation at 37 °C for 10 min, absorbance was measured at 595 nm.

**2.7 Activity recovery**

The standard formula for activity recovery is:

$$Activiyy Recovery \left( \% \right)=\left( \frac{total activity of immobilized enzyme}{total activity of free enzyme} \right)\times100\%$$

**2.8 Selectivity and anti-interference capacity**

Selectivity and anti-interference are key parameters for evaluating sensor performance. As negative controls, 2,4-D, Ca^2+^, Mg^2+^, Zn^2+^, Na^+^, Cl^-^, SO_4_^2-^, PO_4_^3-^, NO_3_^-^, BSA, Ova, imidacloprid, acetamiprid, fipronil, permethrin, cypermethrin, cyhalothrin, and isoprocarb were used to determine the selectivity of AChE@PAH-HOF-based hydrogel disc for chlorpyrifos. For anti-interference ability, the response of AChE@PAH-HOF-based hydrogel to the chlorpyrifos in the presence of the above coexisting interferences was collected. In detail, 25 μL of the above substances were added to microtube and incubated for 30 min. And then, 20 μL ATCh (25 mM) and 20 μL DTNB (0.3 mg mL^-1^) were added in the microtube. The image of hydrogel was analyzed using the commercial software ImageJ. The projected signals (photoluminescence image color) are calculated to obtain Euclidean Distance for evaluating the sensing performance.

**2.9 Real sample detection**

Three actual samples of tap water, apple juice and orange juice were used to test the feasibility of the colorimetric system. The samples were filtered through a 0.22 mm membrane to remove the precipitated substances. The filtered samples were diluted 10 times to attenuate the color interference with the system. The treated samples were spiked with 5 ng mL^-1^, 50 ng mL^-1^ and 500 ng mL^-1^ of chlorpyrifos for chlorpyrifos detection.

**2.10 Chlorpyrifos Degradation in Tomato Plant**

Chlorpyrifos solution was sprayed on tomato seedlings and cultivated for 14 days. At the set point of time (1st, 2nd, 3rd, 4th, 6th, 8th 10th and 14th days), hydrogel discs were placed on the leaf of a tomato plant for 40 min, then, 25 μL ATCh (10 mM) and 25 μL DTNB (0.3 mg mL^-1^) was dispensed onto the hydrogel discs for reacting 40 min. This operation improves pesticide extraction by moistening the surface of the contacting leaves. With the finish of reacting, the image of hydrogel was collected and then analyzed using the commercial software ImageJ to analyze the data information.

**2.11 Enzyme kinetic studies**

The kinetic behavior of AChE@PAH-HOF immobilized acetylcholine ester was investigated and the performance of immobilized acetylcholine ester was evaluated. 25 μL of catalyst, containing AChE@PAH-HOF and AChE, respectively, were added to a mixture containing 100 μL of PBS (10 mM, pH=7.4), 25 μL of water, 50 μL of ATCh at different concentrations, and 50 μL of DTNB (0.3 mg mL^-1^) in a mixed solution. The change in absorbance with time was immediately detected and the kinetic curve was plotted. The kinetic parameters (Km and V_max_) are calculated by the Michaelis-Menten equation:

1/V = (Km / V_max_) (1/^[3]^ + 1/Vmax)

where ^[3]^ stands for the concentration of substrate, V stands for the reaction rate at this concentration of the substrate.

**2.12 ED Calculation**

$$ED=\sqrt{\Delta R^{2}+\Delta B^{2}+\Delta G^{2}}$$

The colorimetric response (ΔR, ΔG, ΔB) of these three materials to water molecules was achieved by subtracting the pre-exposure images from post-exposure images.

**
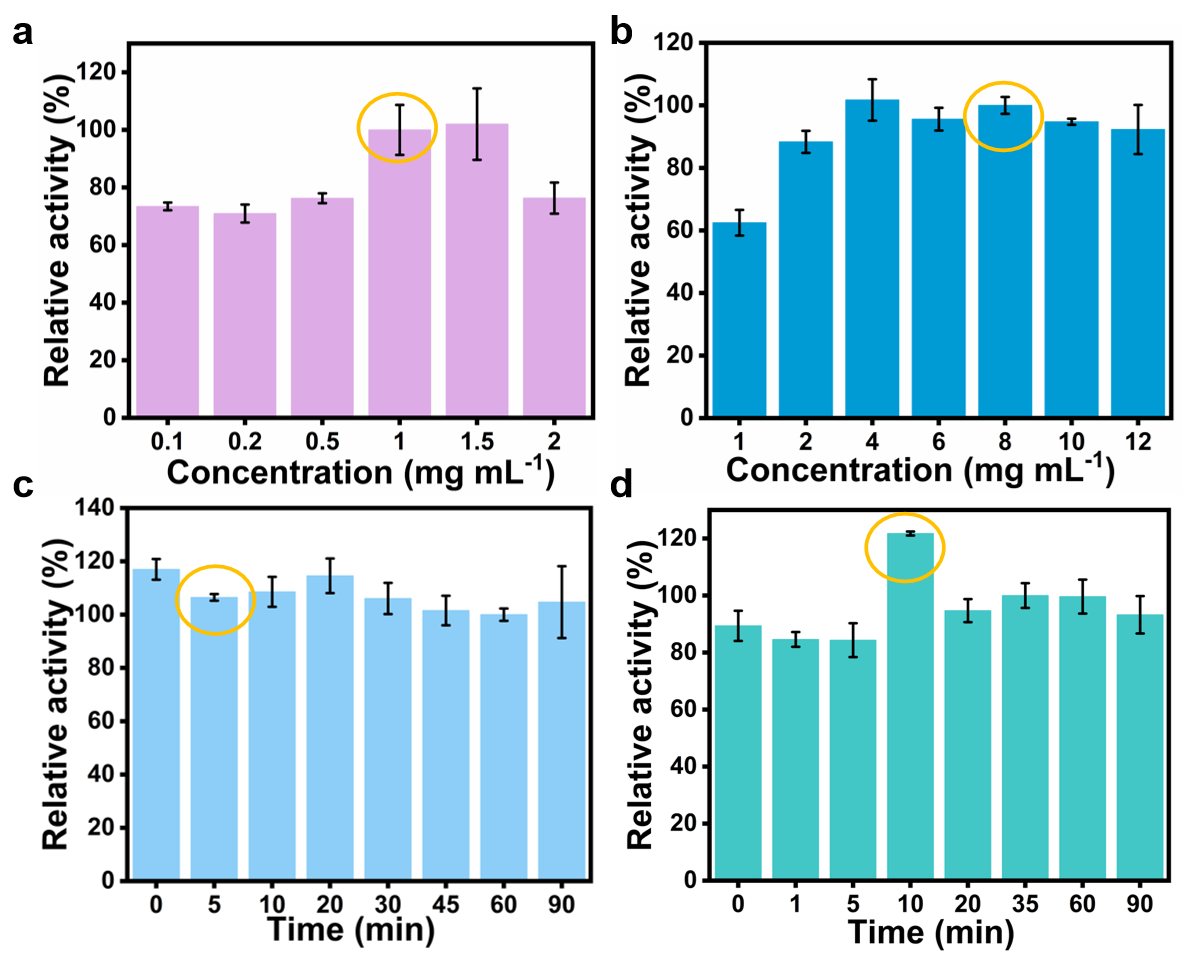
**

**Figure S1** The relative activity of different concentration of PAH (**a**) and H_4_TBAPy (**b**). and the relative activity of different reaction time of PAH with AChE (**c**) and H_4_TBAPy with the mixture of PAH and AChE (**d**).

**
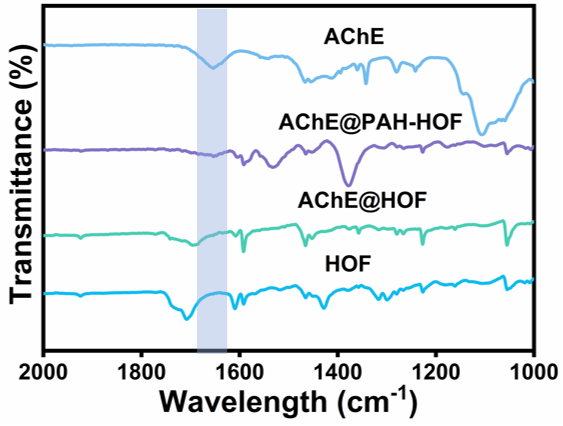
**

**Figure S2** FT-IR spectra of AChE@PAH-HOF, HOF, AChE@HOF and free AChE. The emerging amide I (1659 cm^−1^) bands of AChE@PAH-HOF in Fourier-transform infrared (FT-IR) spectra were assigned to the typical skeleton of the enzyme, further confirming the successful incorporation of AChE.


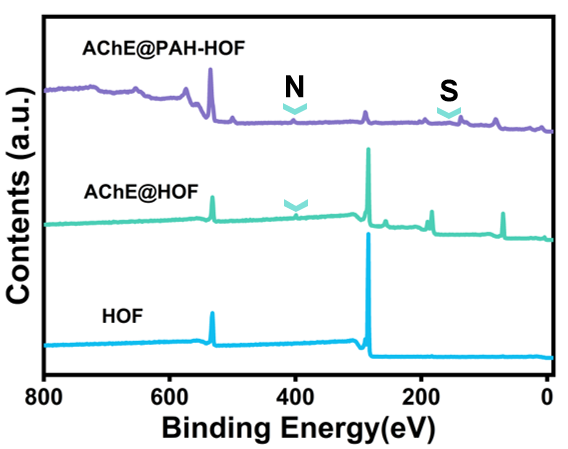


**Figure S3** XPS spectra of AChE@PAH-HOF, AChE@HOF and HOF.


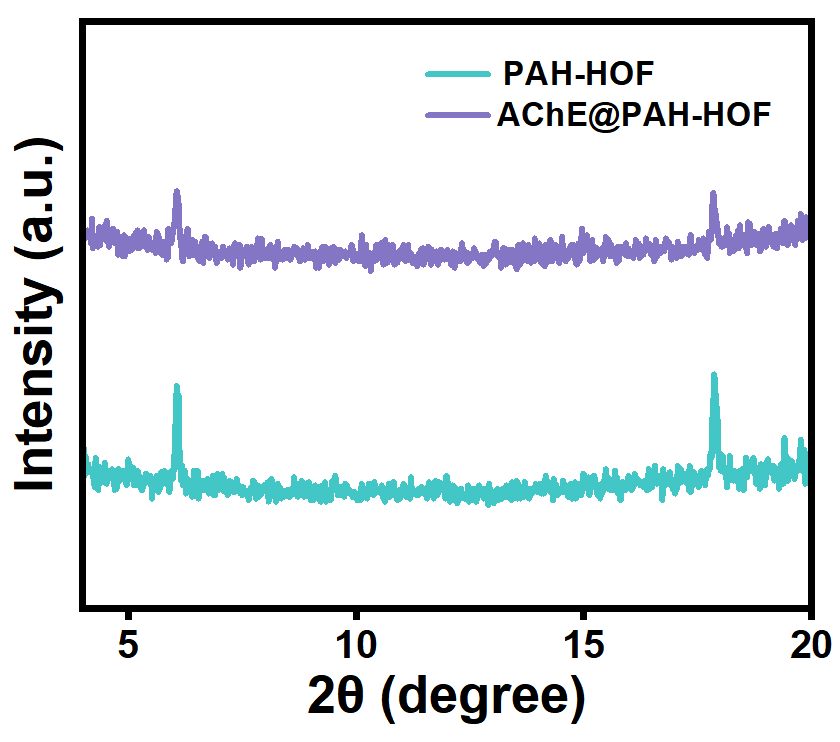


**Figure S4** The XRD pattern of PAH-HOF and AChE@PAH-HOF.

**
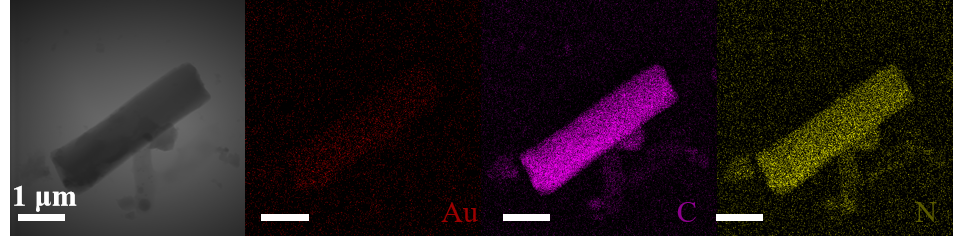
**

**Figure S5** The highly dispersed AuNCs-BSA in AuNCs-BSA@PAH-HOF was observed under the image of TEM and EDS mapping.

**
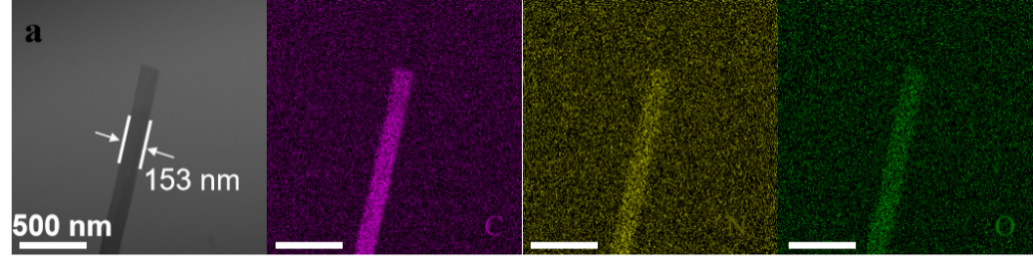
**

**
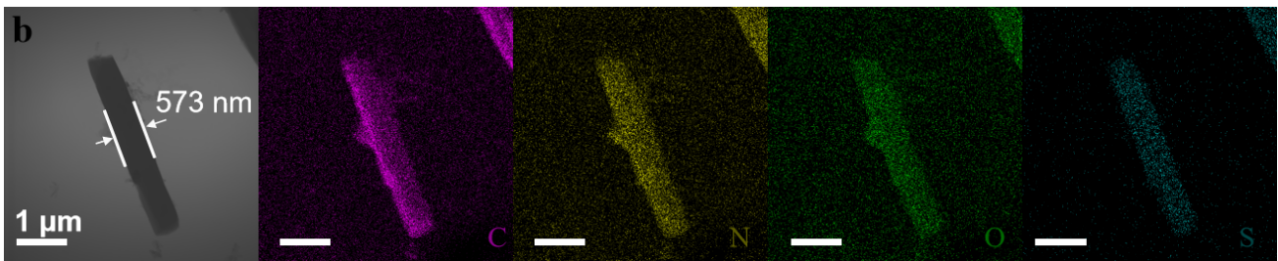
**

**Figure S6** (a) TEM images and EDS mapping of HOF (b) TEM images and EDS mapping of AChE@HOF.


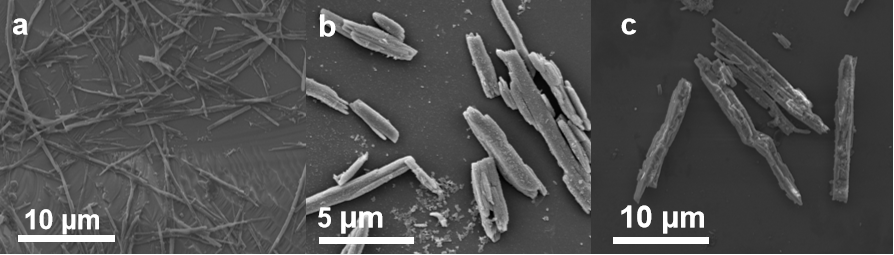


**Figure S7 The SEM image of (a) HOF, (b) AChE@HOF, (c) AChE@PAH-HOF.**


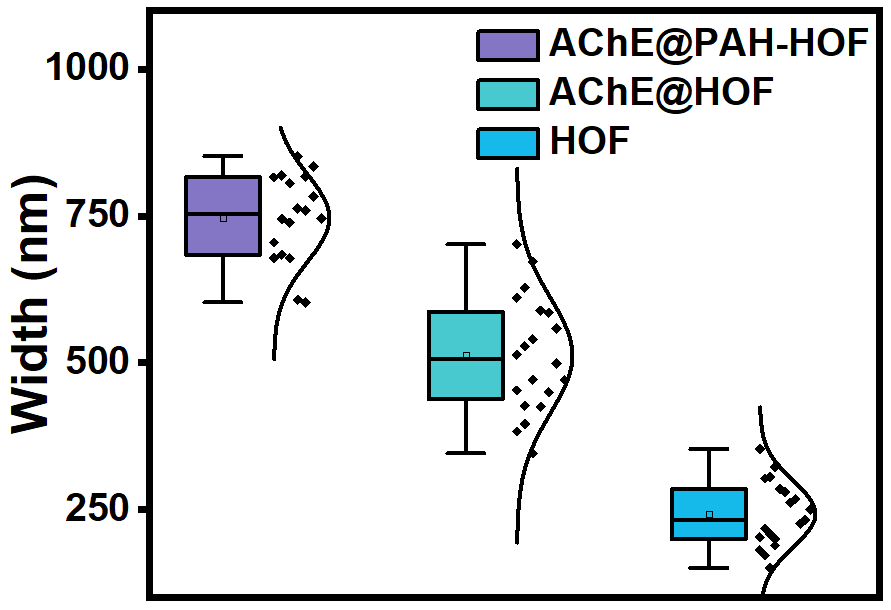


**Figure S8 The width of AChE@PAH-HOF, AChE@HOF, HOF.**

**
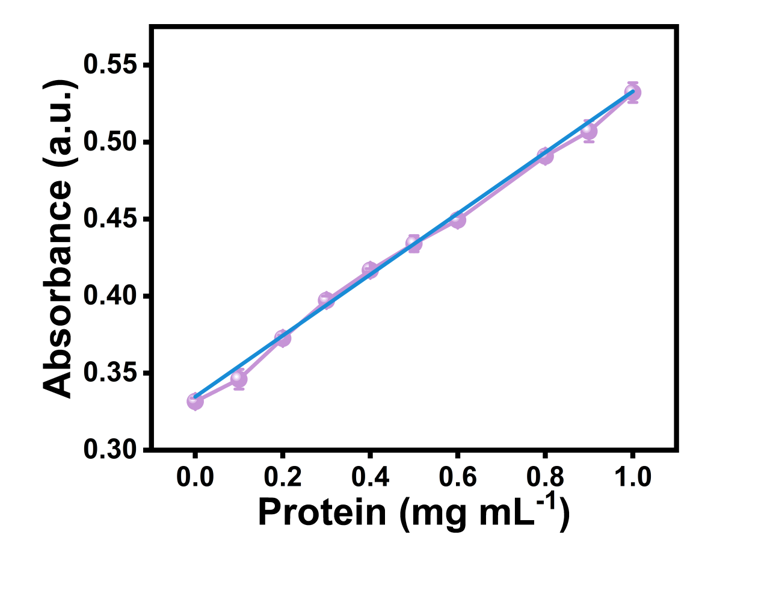
**

**Figure S9** Standard curve based on Bradford protein analysis.


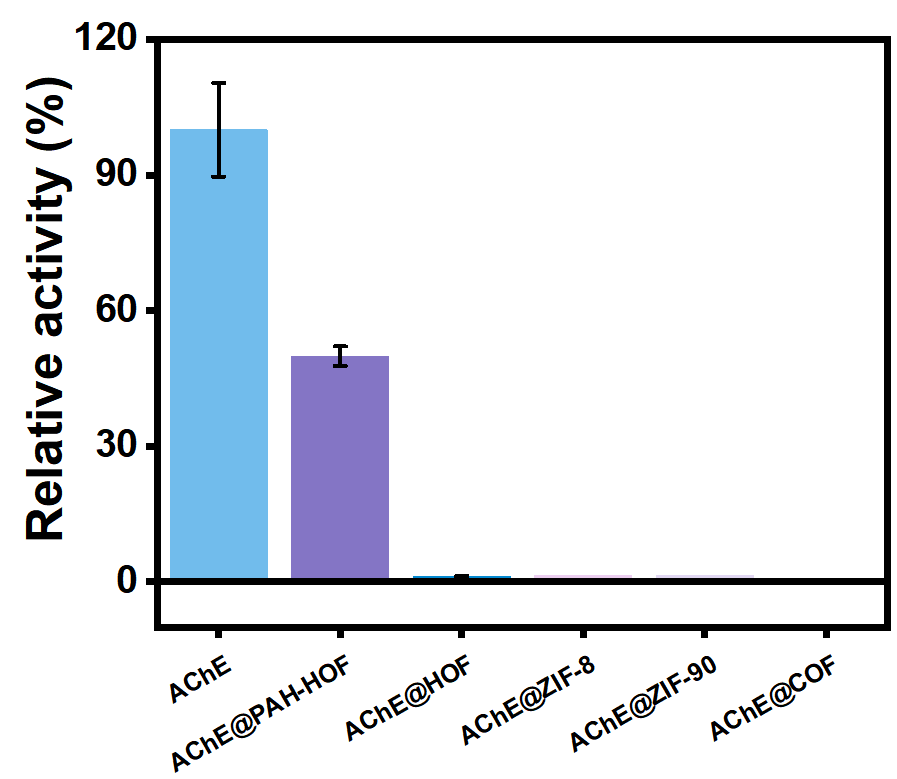


**Figure S10 The relative activity of** AChE, AChE@PAH-HOF, AChE@HOF, AChE@ZIF-8, AChE@ZIF-90, AChE@COF**.**


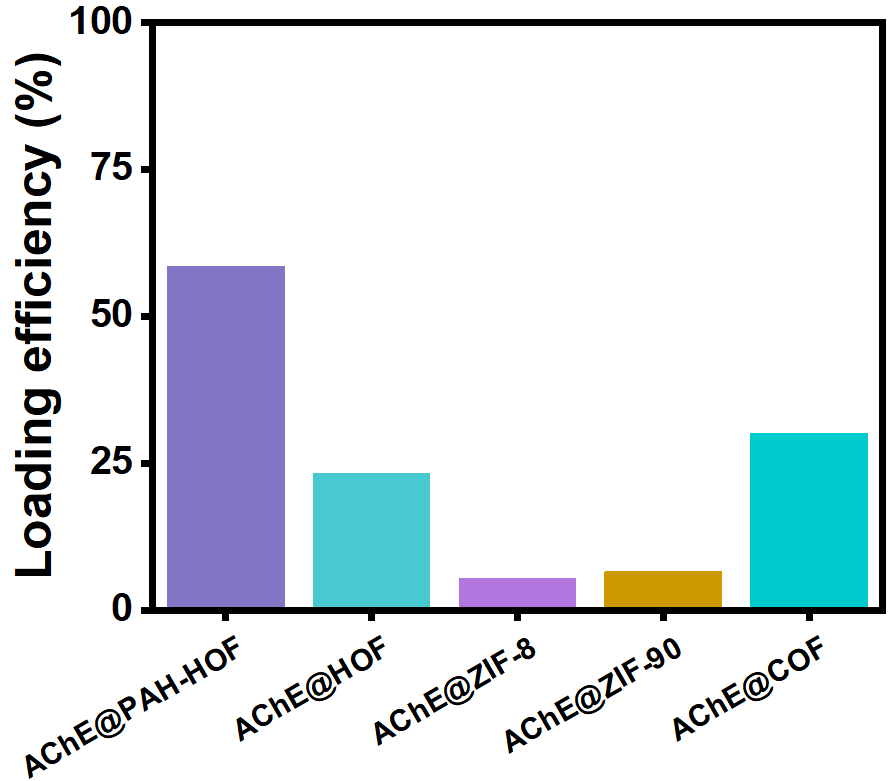


**Figure S11** The loading efficiency of AChE@PAH-HOF, AChE@HOF, AChE@ZIF-8, AChE@ZIF-90, AChE@COF.

**
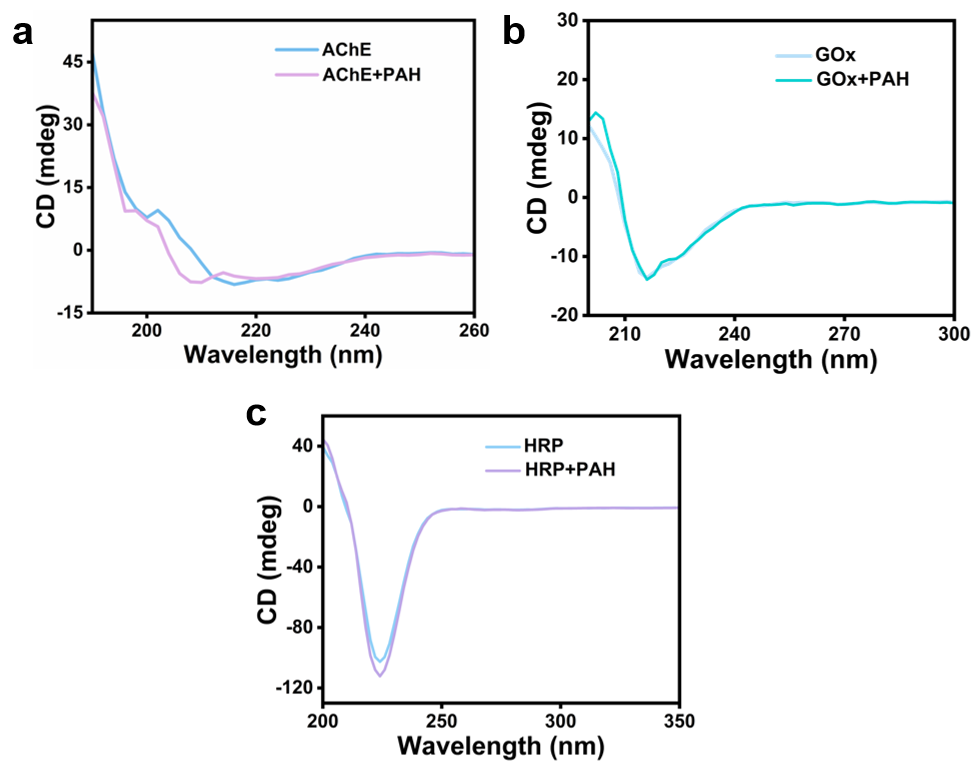
**

**Figure S12** (a) Circular Dichroism (CD) Spectroscopy of AChE and the mixture of AChE and PAH. (b) CD Spectroscopy of GOx and the mixture of GOx and PAH. (c) CD Spectroscopy of HRP and the mixture of HRP and PAH.

**
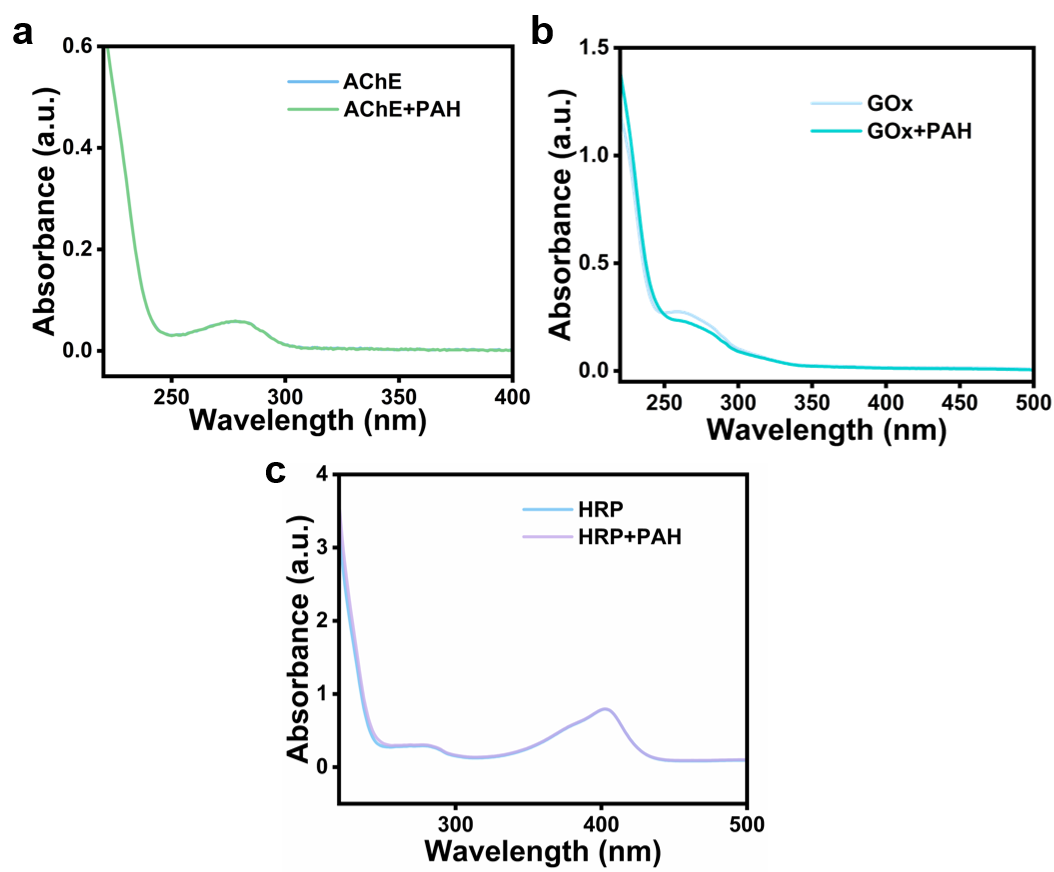
**

**Figure S13** (a)The UV Spectroscopy of AChE and the mixture of AChE and PAH. (b) The UV Spectroscopy of GOx and the mixture of GOx and PAH. (c) The UV Spectroscopy of Peroxidase from HRP and the mixture of HRP and PAH.

**
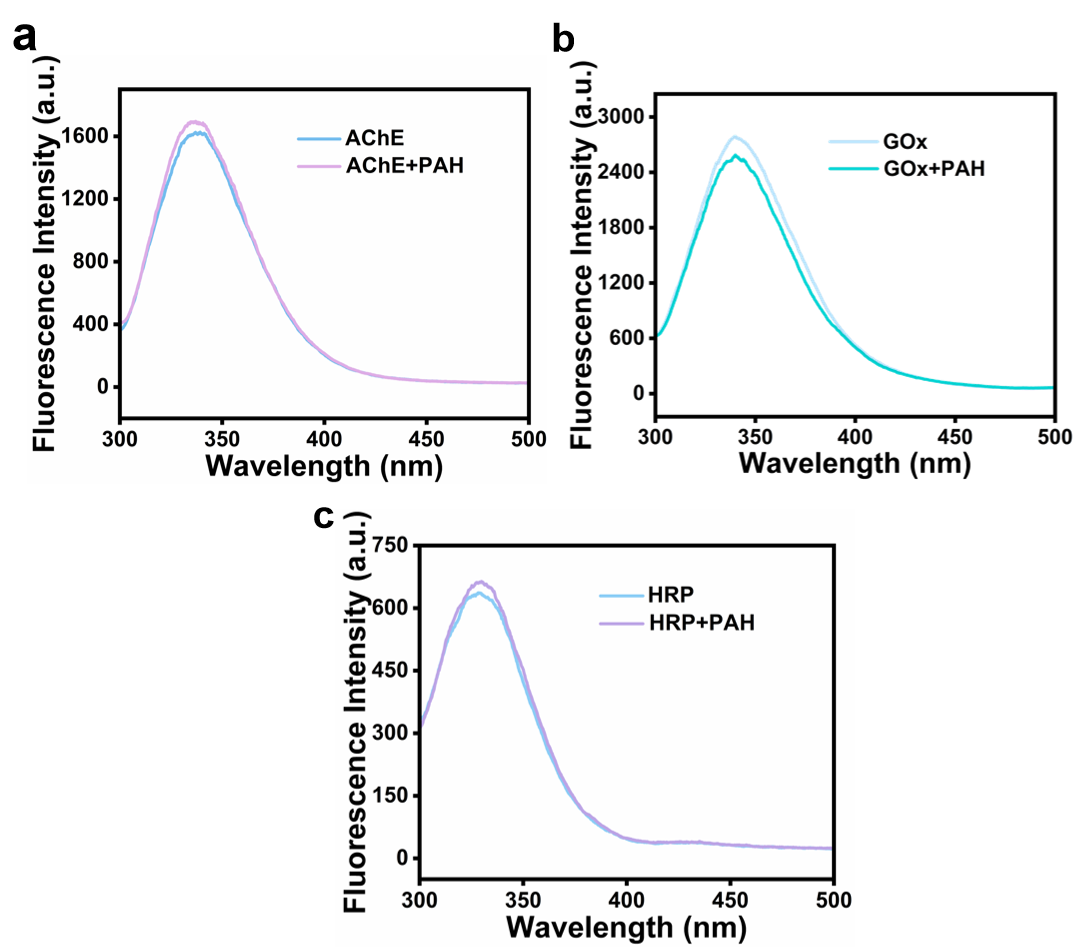
**

**Figure S14** (a) The fluorescence spectroscopy of AChE and the mixture of AChE and PAH. (b) The fluorescence spectroscopy of GOx and the mixture of GOx and PAH. (c) The fluorescence spectroscopy of HRP and the mixture of HRP and PAH.


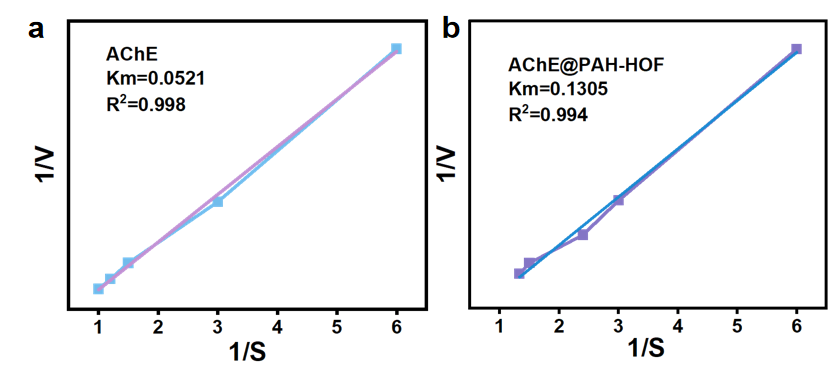


**Figure S15** (a) Lineweaver-Burk plot of AChE. (b) Lineweaver-Burk plot of AChE@PAH-HOF.


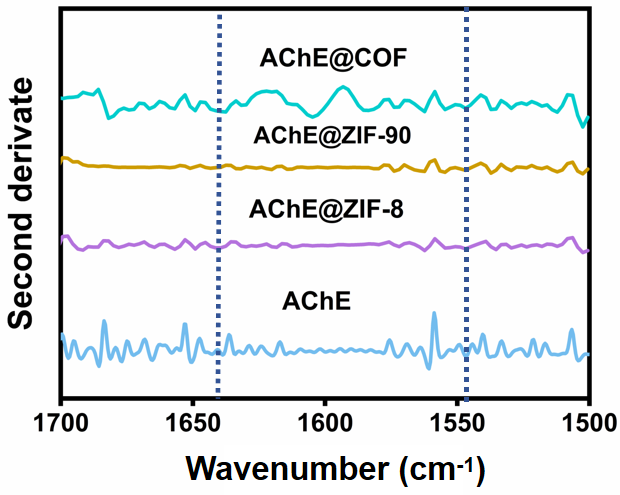


**Figure S16** Second derivative FT-IR spectra.


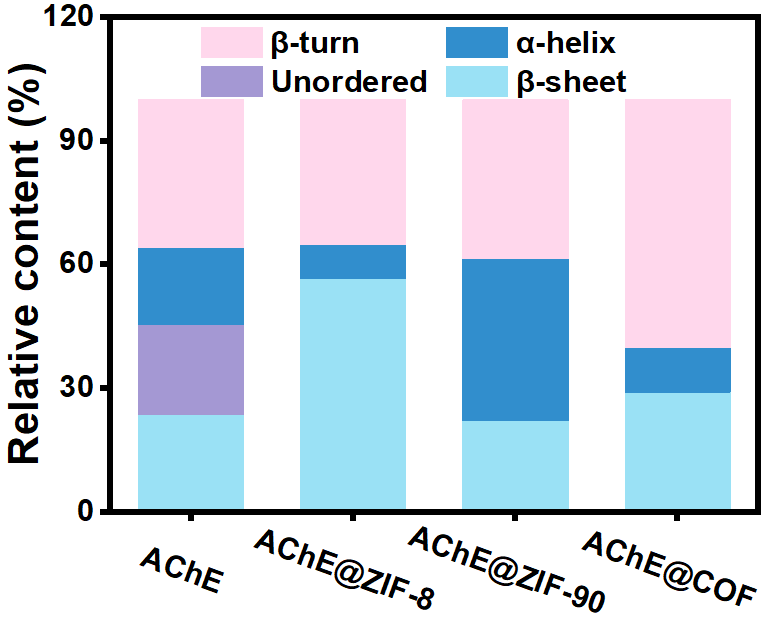


**Figure S17** Secondary structure contents of AChE, AChE@ZIF-8, AChE@ZIF-90 and AChE@COF.

**
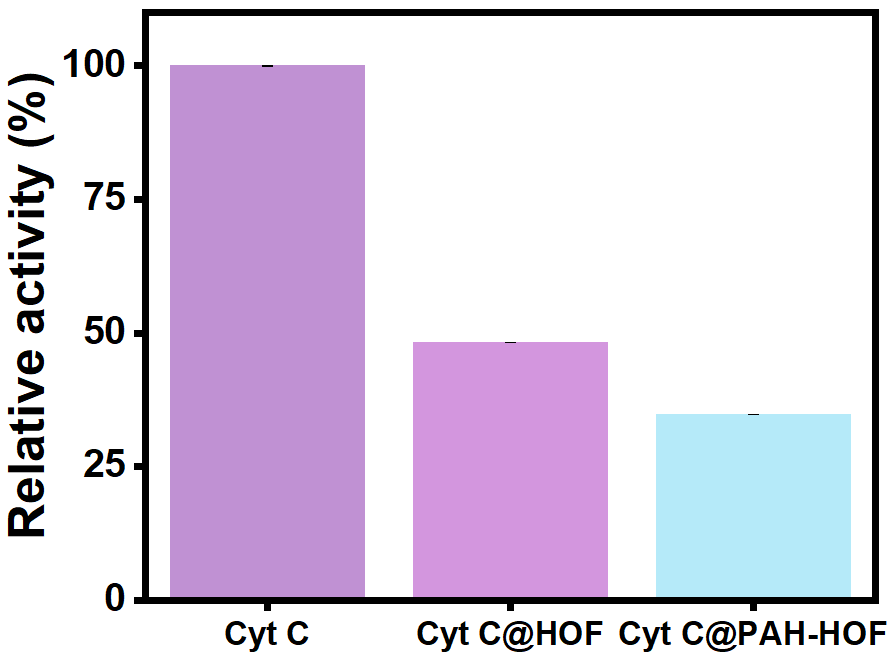
**

**Figure S18** The relative activity of Cyt C, CytC @HOF, Cyt C@PAH-HOF.

**
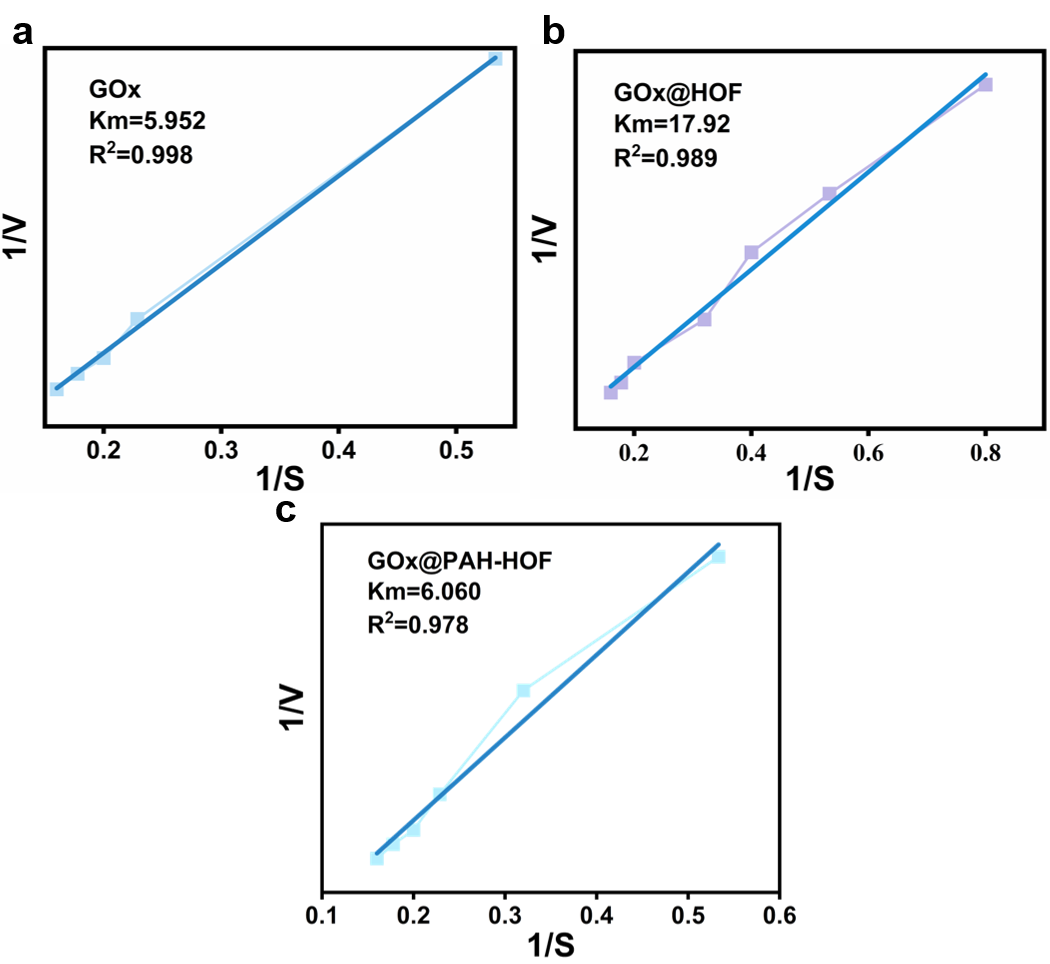
**

**Figure S19** (a) Lineweaver-Burk plot of GOx. (b) Lineweaver-Burk plot of GOx@HOF. (c) Lineweaver-Burk plot of GOx@PAH-HOF.

**
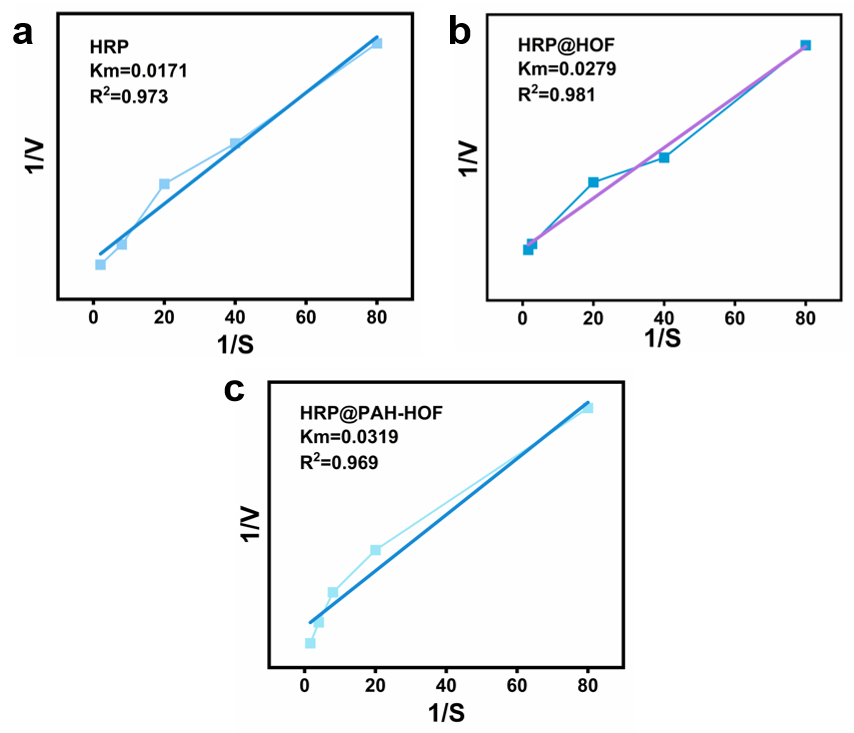
**

**Figure S20** (a) Lineweaver-Burk plot of HRP. (b) Lineweaver-Burk plot of HRP@HOF. (c) Lineweaver-Burk plot of HRP@PAH-HOF.

**
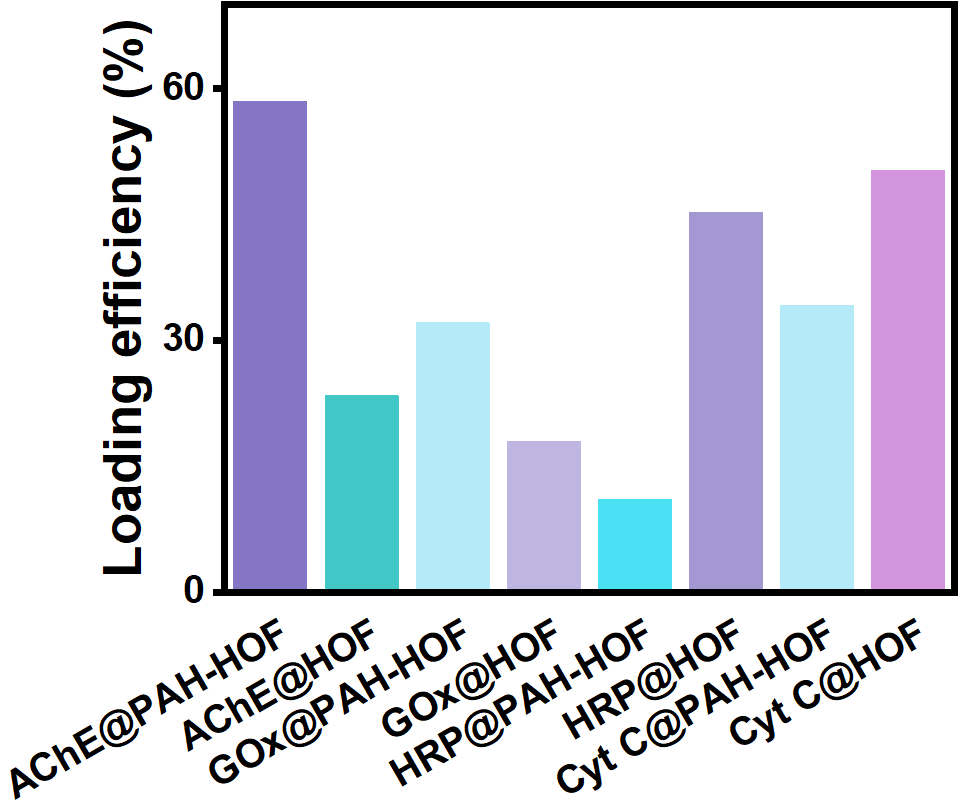
**

**Figure S21** The loading efficiency of different enzyme.

**
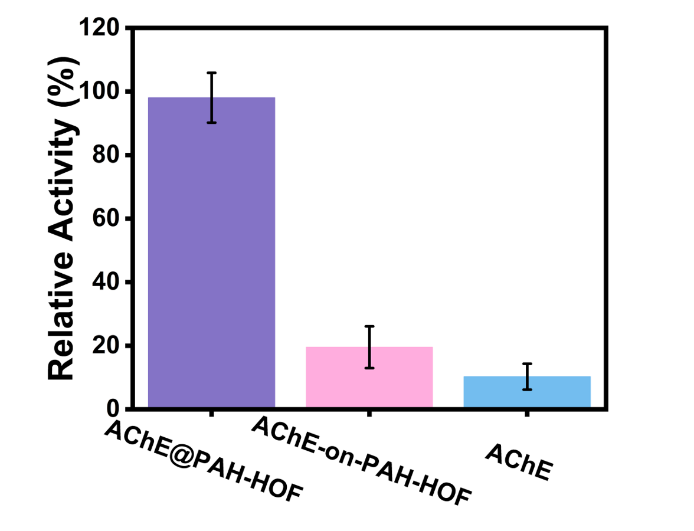
**

**Figure S22** The relative activity of different composites after trypsin treatment for 24h.

**
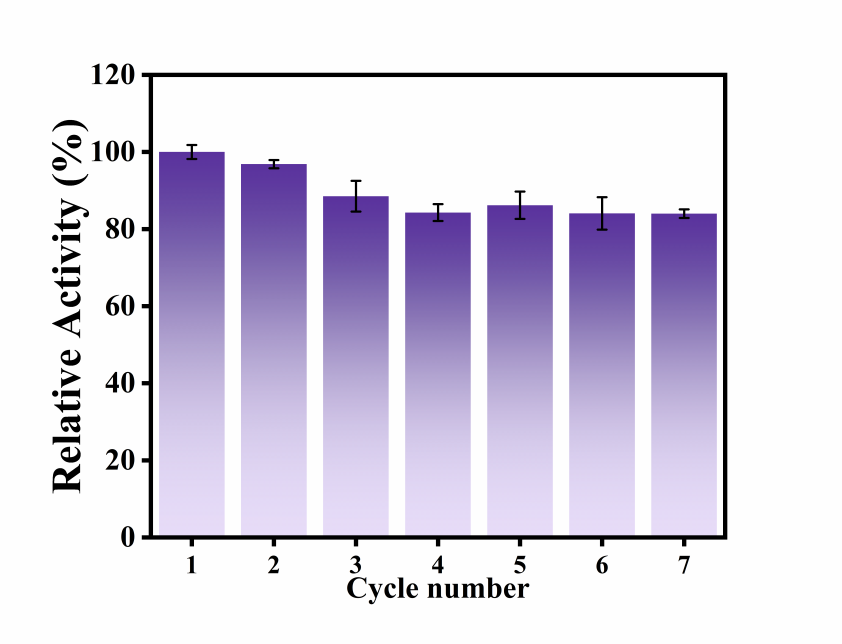
**

**Figure S23** The cyclic stability of AChE@PAH-HOF.

**
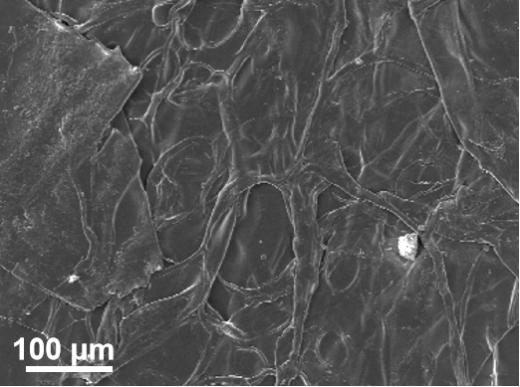
**

**Figure S24** The SEM image of glycerol-sodium alginate hydrogel.

**
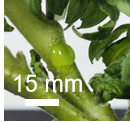
**

**Figure S25** The optical image of hydrogel disc in tomato stem.

**
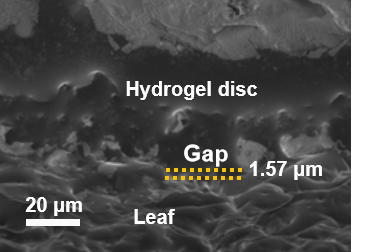
**

**Figure S26** Cross-sectional SEM image of a hydrogel applied to a tomato-plant leaf showing a high degree of hydrogel-disc conformability**.**

**
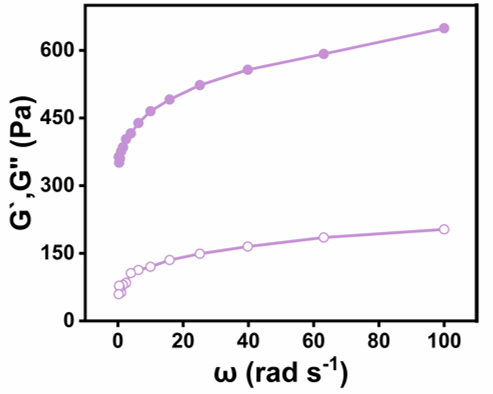
**

**Figure S27** The rheological properties test. The result showed that the softness of the hydrogel disc enables conformal contact with plant leaf surfaces.

**
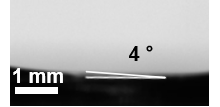
**

**Figure S28** Water contact angle measurement. A small water contact angle indicates the hydrophilic nature of the hydrogel.





**Figure S29** Water retention capacity of hydrogel discs with different levels of glycerol addition.

**
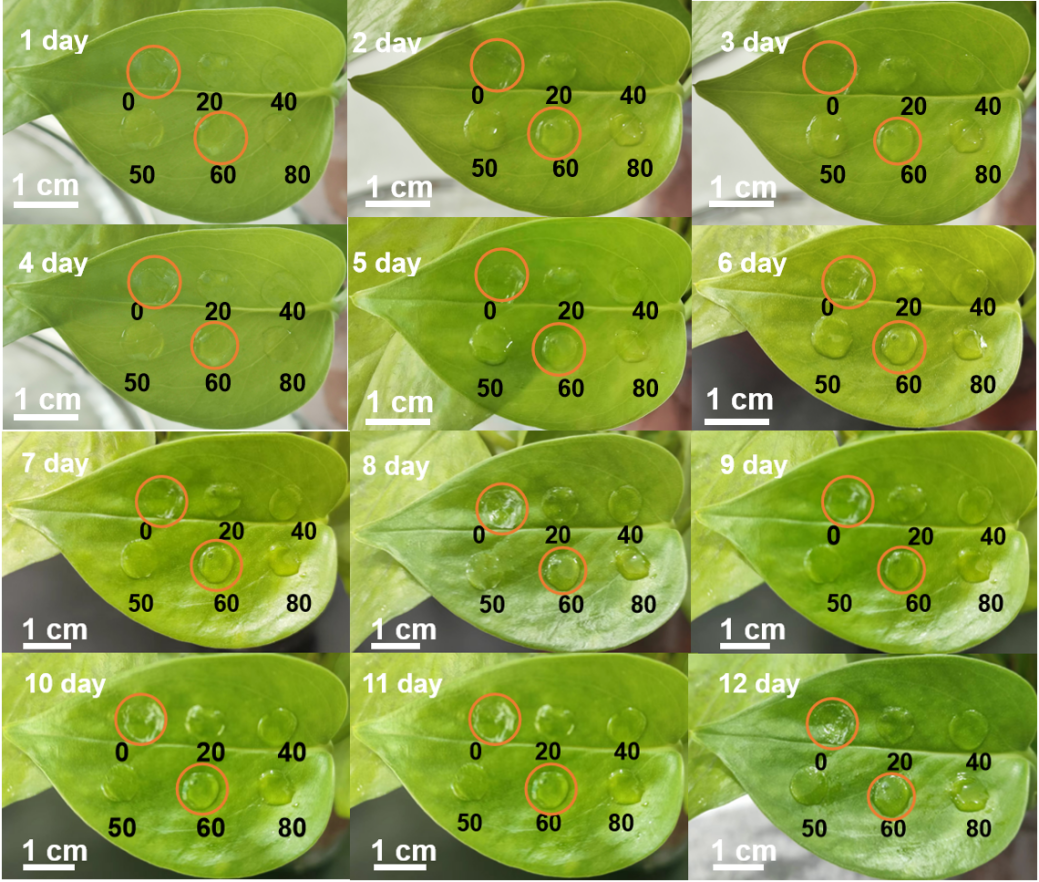
**

**Figure S30** Morphological changes of glycerol-sodium alginate hydrogel on leaf surface in 12 days at room temperature. Hydrogel discs with varying glycerol content were placed on plants, and their morphological changes were observed over several days. Morphological analysis reveals structural collapse in glycerol-free sodium alginate gels, whereas the incorporation of glycerol significantly improves structural integrity.

**

**

**Figure S31** The weight retention behavior of SA hydrogels and G-SA hydrogels under different temperatures (45 ℃, 25 ℃).

**
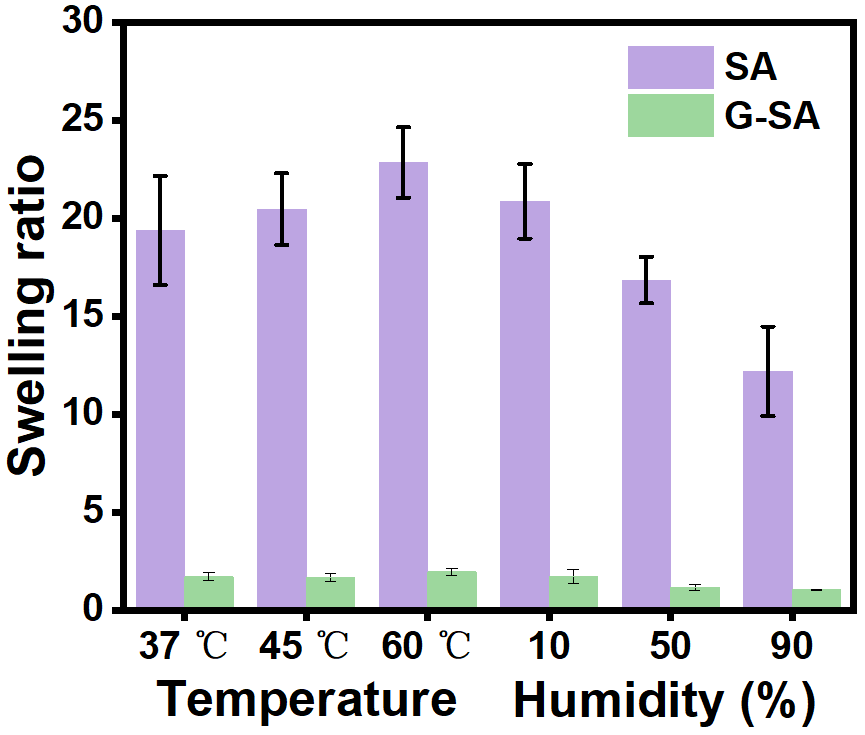
**

**Figure S32** The swelling ratio of G-SA and SA at different humidity and temperature.

**
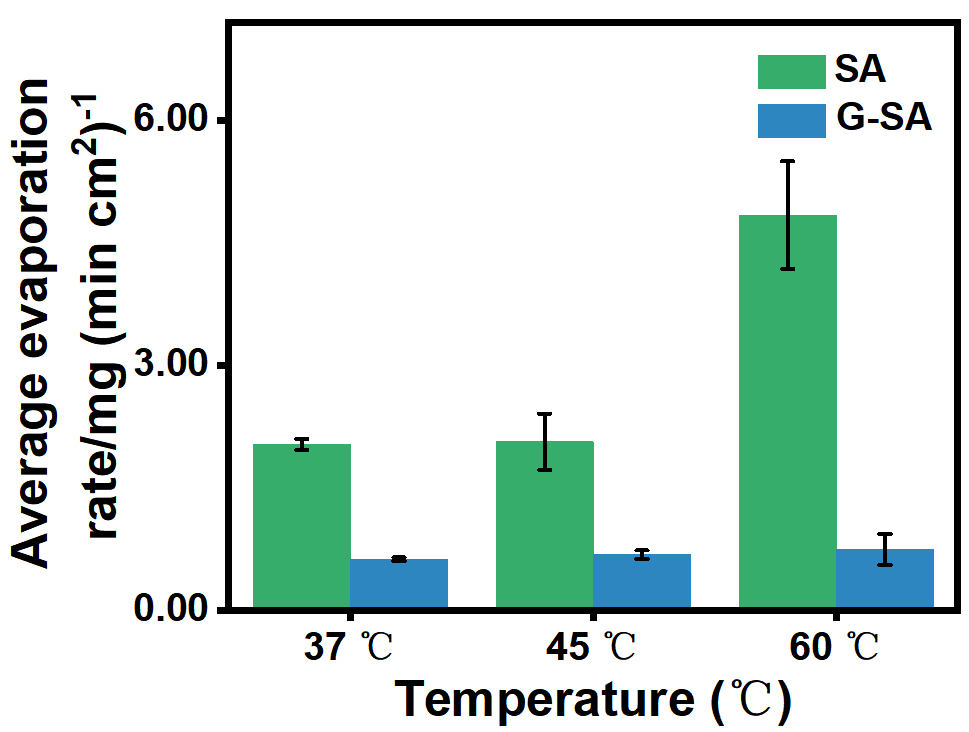
**

**Figure S33** The evaporation of G-SA and SA at different temperatures.

**
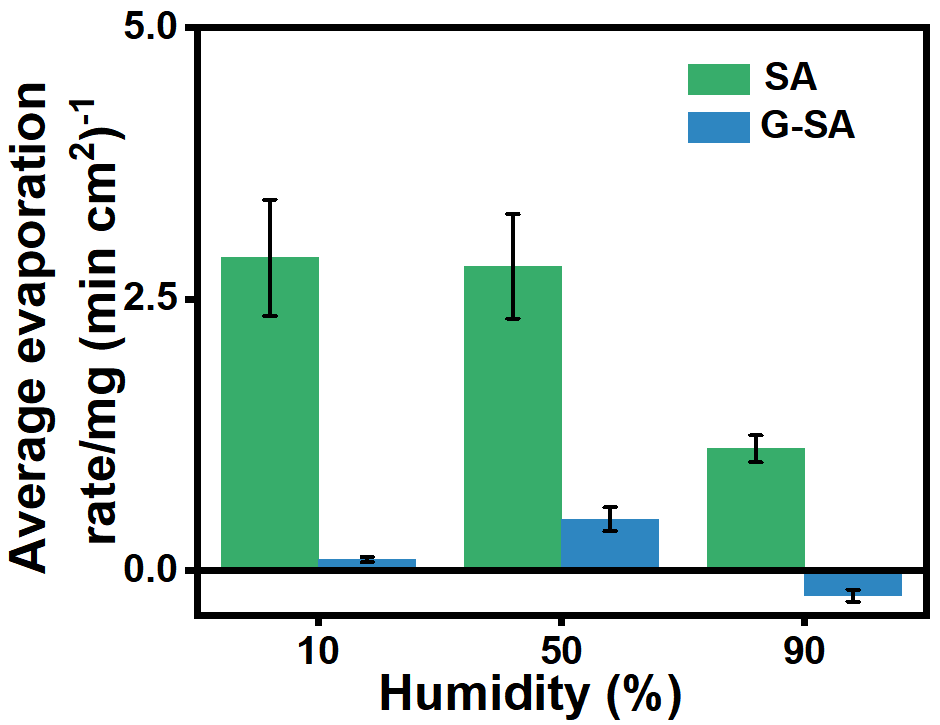
**

**Figure S34** The evaporation of G-SA and SA at different humidity levels.

**
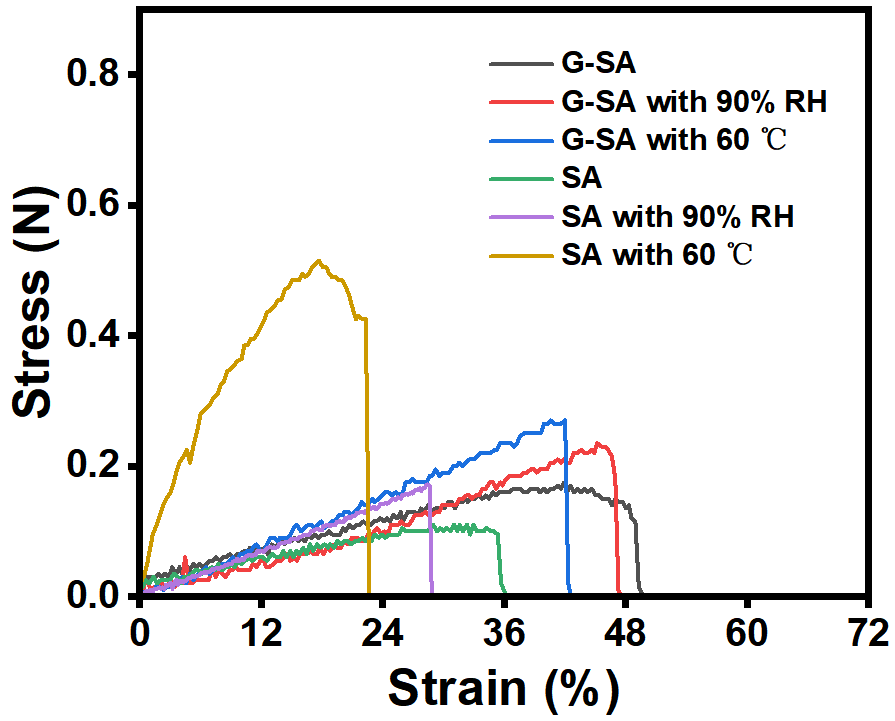
**

**Figure S35** The stress–strain curves of G-SA and SA in high temperature and humidity.

**
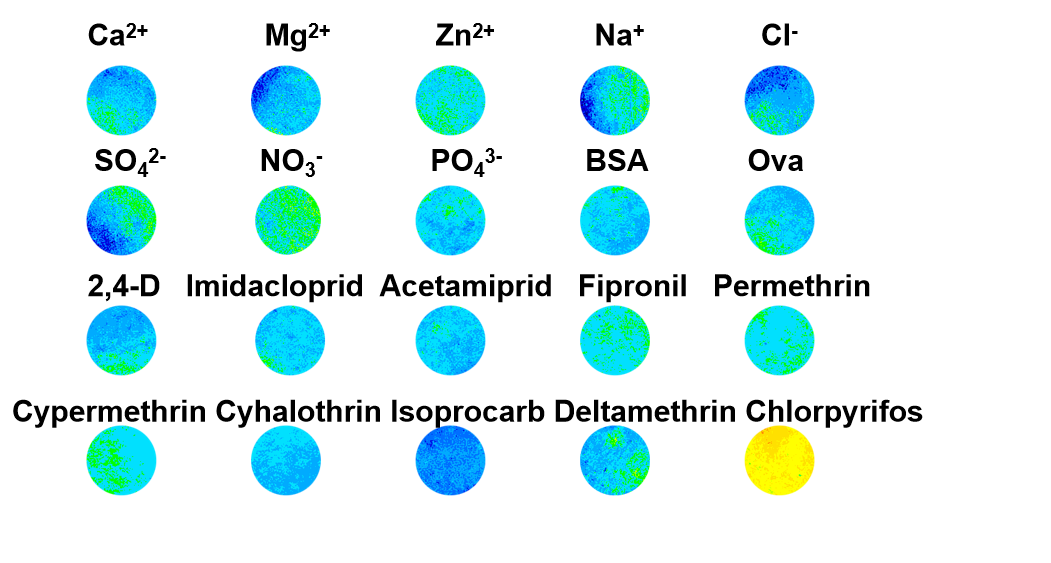
**

**Figure S36** Selectivity performance of the hydrogel discs.

**
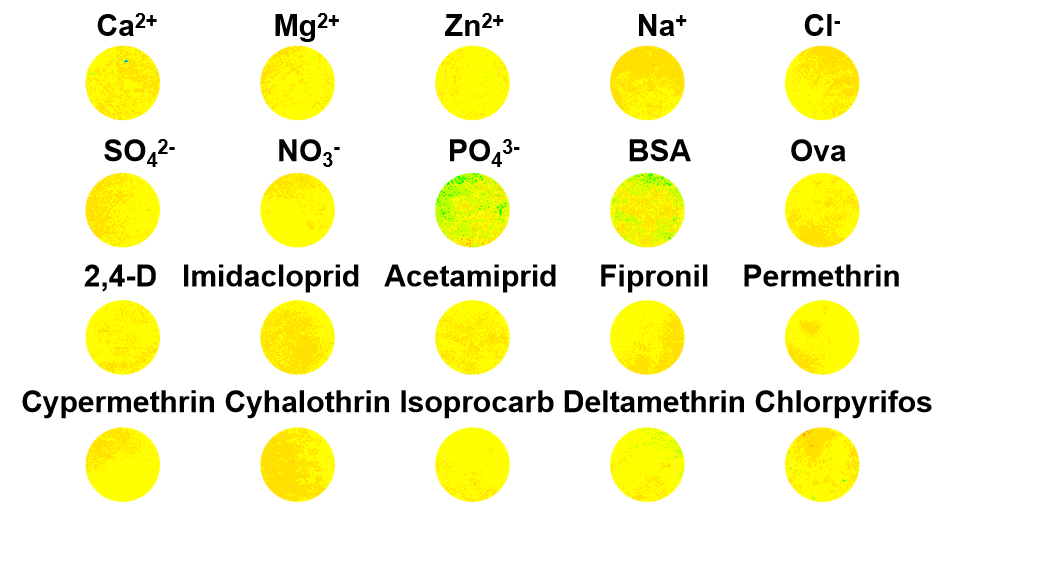
**

**Figure S37** Anti-interference performance of the hydrogel discs.

**

**

**Figure S38** Bland-Altman analysis was used to evaluate the agreement between HPLC and established biosensor.

**Table S1** Comparison of enzyme loading of HOF immobilized enzymes.

| HOF | Enzyme | Enzyme loading | Ref |
| --- | --- | --- | --- |
| Bio-HOF | FAOx | 2.9% | ^[4]^ |
| HOF-101 | BSA | 24.3% | ^[2]^ |
| TNU-14 | CAT | 17.5% | ^[5]^ |
| HOF-100 | HRP | 36.6% | ^[6]^ |
| PAH-HOF | AChE | 58.5% | This work |

**Table S2** Comparison of recovery rate between PWS and HPLC.

| **Sample** | **Spiked**  **（ng mL^-1^）** | **Found (ng mL^-1^)**  **Recovery** | | **Recovery**  **(%)** | **RSD**  **(n=3, %)** |
| --- | --- | --- | --- | --- | --- |
|  |  | **This work** | **HPLC** |  |  |
| **Tap Water** | **5** | **5.10** | **-** | **102.00** | **4.93** |
|  | **50** | **47.95** | **48.00** | **95.90** | **9.72** |
|  | **500** | **498.09** | **501.00** | **99.62** | **2.11** |
| **Orange juice** | **5** | **4.98** | **-** | **99.60** | **3.02** |
|  | **50** | **49.38** | **53.30** | **98.76** | **0.67** |
|  | **500** | **533.9** | **464.00** | **106.78** | **7.53** |
| **Apple juice** | **5** | **5.08** | **-** | **101.60** | **2.96** |
|  | **50** | **47.95** | \| **53.60** \| \| --- \| | **95.90** | **4.45** |
|  | **500** | **504.18** | **522.00** | **100.84** | **4.67** |

[1] S. Liu, Y. Sun, *Angew. Chem. Int. Edit.* **2023**, *62*, e202308562.

[2] G. S. Chen, S. M. Huang, Y. Shen, X. X. Kou, X. M. Ma, S. Y. Huang, Q. Tong, K. L. Ma, W. Chen, P. Y. Wang, J. Shen, F. Zhu, G. F. Ouyang, *Chem-Us* **2021**, *7*, 2722-2742.

[3] R. Umapathi, B. Park, S. Sonwal, G. M. Rani, Y. J. Cho, Y. S. Huh, *Trends Food Sci Tech* **2022**, *119*, 69-89.

[4] P. Wied, F. Carraro, J. M. Bolivar, C. J. Doonan, P. Falcaro, B. Nidetzky, *Angew. Chem. Int. Edit.* **2022**, *134,* e202117345.

[5] J. Xu, M. X. Zuo, H. Y. Zhang, Z. Y. Di, C. P. Li, *Inorg. Chem. Front.* **2025**, *12*, 2459-2467.

[6] W. Huang, H. T. Yuan, H. S. Yang, X. M. Ma, S. Y. Huang, H. J. Zhang, S. M. Huang, G. S. Chen, G. F. Ouyang, *Nat. Commun* **2023**, *14*, 3644.
